# fullfact: an R package for the analysis of genetic and maternal variance components from full factorial mating designs

**DOI:** 10.1002/ece3.1943

**Published:** 2016-02-14

**Authors:** Aimee Lee S. Houde, Trevor E. Pitcher

**Affiliations:** ^1^ Department of Biological SciencesUniversity of WindsorWindsorOntarioN9B 3P4Canada; ^2^ Great Lakes Institute for Environmental ResearchUniversity of WindsorWindsorOntarioN9B 3P4Canada

**Keywords:** Additive genetic effects, compatible genes, genetic architecture, good genes, mate choice, maternal effects, nonadditive genetic effects, North Carolina II design, statistical power

## Abstract

Full factorial breeding designs are useful for quantifying the amount of additive genetic, nonadditive genetic, and maternal variance that explain phenotypic traits. Such variance estimates are important for examining evolutionary potential. Traditionally, full factorial mating designs have been analyzed using a two‐way analysis of variance, which may produce negative variance values and is not suited for unbalanced designs. Mixed‐effects models do not produce negative variance values and are suited for unbalanced designs. However, extracting the variance components, calculating significance values, and estimating confidence intervals and/or power values for the components are not straightforward using traditional analytic methods. We introduce fullfact – an R package that addresses these issues and facilitates the analysis of full factorial mating designs with mixed‐effects models. Here, we summarize the functions of the fullfact package. The observed data functions extract the variance explained by random and fixed effects and provide their significance. We then calculate the additive genetic, nonadditive genetic, and maternal variance components explaining the phenotype. In particular, we integrate nonnormal error structures for estimating these components for nonnormal data types. The resampled data functions are used to produce bootstrap‐*t* confidence intervals, which can then be plotted using a simple function. We explore the fullfact package through a worked example. This package will facilitate the analyses of full factorial mating designs in R, especially for the analysis of binary, proportion, and/or count data types and for the ability to incorporate additional random and fixed effects and power analyses.

## Introduction

The full factorial mating design (also known as the North Carolina II design), such that dams and sires are mated in all possible pairwise combinations, has the advantage of estimating the genetic variance (*V*_*G*_) and environmental variance (*V*_*E*_) components of the phenotypic variance (*V*_*P*_) of offspring traits (Lynch and Walsh 1998, p. 598). Genetic variance can be further divided into additive genetic variance (*V*_*A*_, effects of gene substitution) and nonadditive genetic variance (*V*_*N*_, effects of dominance, the interactions between alleles, and effects of epistasis, the interactions between loci). Indeed, the full factorial mating design is one of the best methods to simultaneously estimate phenotypic additive and nonadditive genetic variance (dominance variance because epistasis variance is assumed to be of negligible importance) (Lynch and Walsh 1998; Neff and Pitcher 2005; Neff et al. 2011). This design has been used in numerous studies to produce at least 100 estimates of genetic variance (Puurtinen et al. 2009). Environmental variance sources can include experimental treatment differences, developmental differences, and maternal environmental differences (Lynch and Walsh 1998). In addition, the phenotypic variance of traits can also be composed of genotype by environment variance (*V*_*G*×*E*_, effects of the interactions between genotypes and environments), which is of interest to the study of local adaptation, inbreeding depression, outbreeding depression, and domestication (Allendorf et al. 2013). Using a common experimental environment for rearing families may reduce some sources of environmental variance to better estimate genetic variance (Lynch and Walsh 1998).

The genetic and maternal variance of traits is of interest for studying evolutionary potential of the traits. Additive genetic variance can be used to predict the response to selection pressures. However, nonadditive genetic variance can be converted to additive genetic variance if there is a change in allele frequency, for example, during a bottleneck because of random genetic drift (Carson 1990). Also, the maternal variance (maternal genetic and environmental variance) of traits can influence the evolutionary potential of the species based on the correlation between maternal and offspring traits and the phenotypic plasticity of female traits (Kirkpatrick and Lande 1989; Mousseau and Fox 1998; Räsänen and Kruuk 2007). Therefore, estimating the additive, nonadditive, and maternal variance contributions of traits is fully needed to understand evolutionary potential.

## Full Factorial Analysis and Issues

Full factorial mating designs, such that *n* dams and *n* sires are mated in all possible pairwise combinations (*n *× *n*), have traditionally been analyzed using two‐way analysis of variance (ANOVA) with mean squares (Lynch and Walsh 1998, p. 600). The phenotypic variance of offspring traits is composed of measurements from the families, and this variance is partitioned into components for the dam (*V*_*D*_, maternal genetic and environmental variance), the sire (*V*_*S*_, paternal genetic variance), and the dam by sire interaction (*V*_*D*×*S*_, nonadditive genetic variance). Assuming the effects of epistasis are of negligible importance, the additive genetic variance (*V*_*A*_) component is calculated as four times the sire (*V*_*S*_), the nonadditive genetic variance (*V*_*N*_) component as four times the dam by sire interaction (*V*_*D*×*S*_), and the maternal variance component (*V*_*M*_) as the dam (*V*_*D*_) – sire (*V*_*S*_) (Lynch and Walsh 1998, p. 603). When there is epistasis, those variance components will be overestimated and this may explain why the percentage of phenotypic variance explained by the components can add up to more than 100% in certain cases (Neff and Pitcher 2005).

There are a couple of issues that can arise from using two‐way ANOVAs to analyze full factorial mating designs: (1) the possibility of negative variance components, for example, insufficient data; and (2) the influence of unbalanced sample sizes, for example, different family sizes or missing family values (Lynch and Walsh 1998, p. 779; Graham and Edwards 2001; Neff and Pitcher 2005). The estimation of the variance components as random effects using maximum likelihood (ML) or restricted maximum likelihood (REML) does not produce negative variance components and is not sensitive to unbalanced sample sizes. The mixed‐effects models package lme4 (Bates et al. 2015) for the statistical program R (R Development Core Team 2015) is suited for full factorial mating designs because the functions can model several random and fixed effects using ML and REML.

Yet, we developed the fullfact package because of the analytical difficulty of extracting variance components and confidence intervals (or significance values and power values) for these components. In particular, the expansion of data to the individual‐level to properly estimate variance components (Puurtinen et al. 2009) is underutilized. This expansion can be time‐consuming if performed by hand, and there is no function (until now) to expand the data. In addition, producing confidence intervals for the variance components is underutilized because it can be time‐consuming and requires higher level coding to resample the data (typically 1000 times) and apply a new model to each of the data sets. Confidence intervals would be useful for visualization of the components and also have the advantage of statistically comparing pairwise groups, such as populations (Houde et al. 2013, 2015).

We also developed the fullfact package to incorporate fixed effects and nonnormal models, which are also underutilized for these types of analyses. For example, previous full factorial analyses have used normal models for proportion type data (e.g., Pitcher and Neff 2007). The fullfact package more carefully examines the parameter space and can conduct other helpful analyses, including the assay of statistical power (both *a priori* using simulated data based on the literature and *post hoc*) for full factorial mating designs. This study summarizes and incorporates recent advances for extracting the variance components of fixed effects (e.g., Snijders and Bosker 1999; Nakagawa and Schielzeth 2013) and from nonnormal models (that do not provide the residual variance found in normal models, so the residual variance needs to be added) (Nakagawa and Schielzeth 2010, 2013). Finally, we provide an example using the fullfact package for Chinook salmon (*Oncorhynchus tshawytscha*) nonnormal early‐life survival data using an 11 × 11 factorial which was originally analyzed by Pitcher and Neff (2007).

## The Fullfact Package

**Figure 1:**
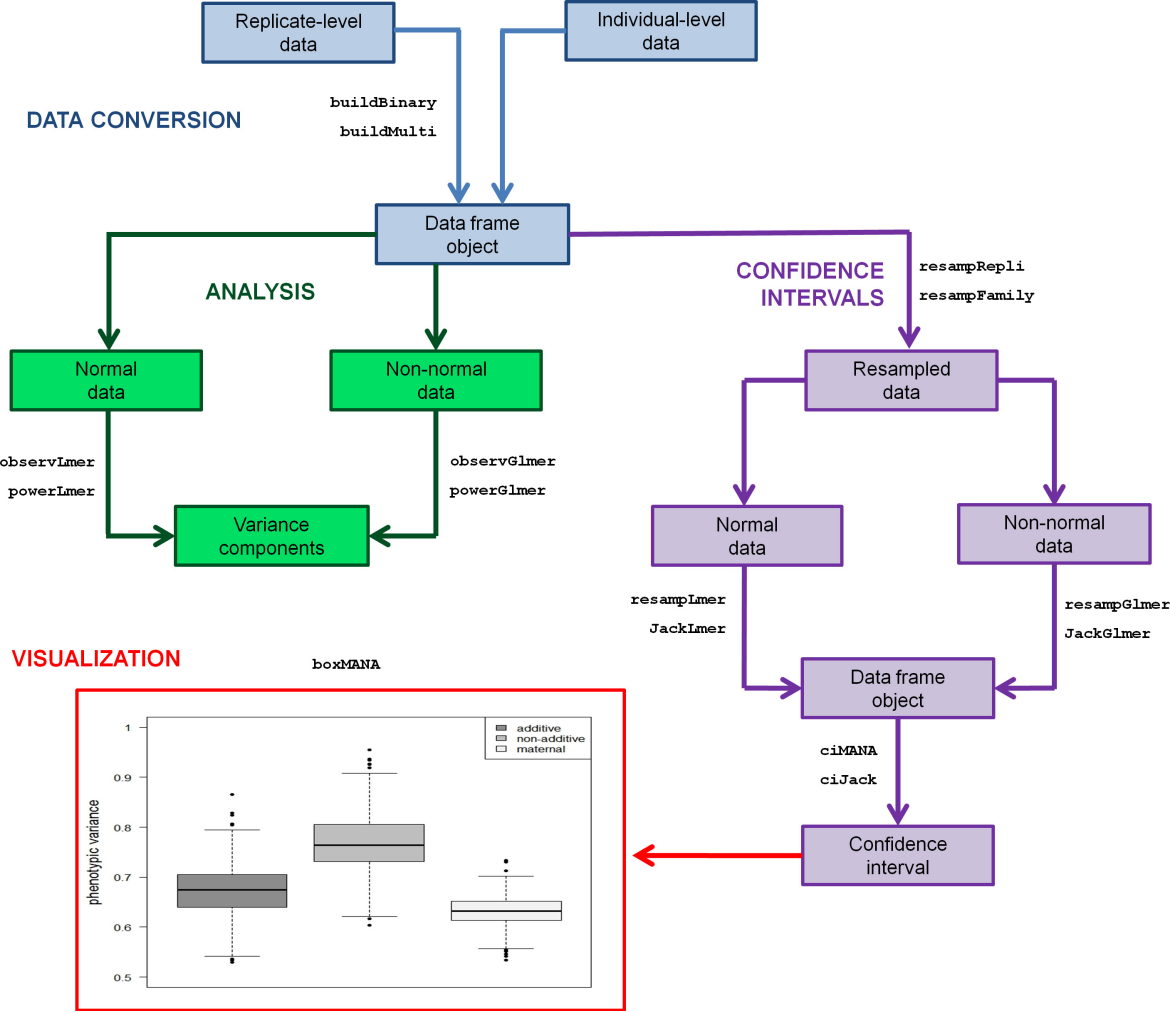
The workflow stages of the fullfact package, highlighting its main analytical functions and simple plotting function.

For direct installation into R, the stable release version of fullfact is available from CRAN (http://CRAN.R-project.org). Installation of the mixed‐effects models package lme4 (Bates et al. 2015) is a prerequisite for the functionality of the fullfact package. Installation of the analysis of factorial experiments package afex (Singmann et al. 2015) may also be required if examining the significance of fixed effects. Functions within the fullfact package were produced based on three levels of complexity for the experimental design: (1) simple (designated by no number) for the standard model, that is, containing random effects for dam, sire, and dam by sire; (2) advanced (designated by the number 2) for the standard model with the options of including additional random effects for one position (e.g., tank) and/or one block effect (e.g., several blocks of 2 × 2 factorial matings); and (3) expert (designated by the number 3) for the standard model with the ability of the user to include additional fixed and random effects, such as a model including environment treatments and their interactions (e.g., Evans et al. 2010). The package was developed with four workflow stages in mind (Fig. 1): (1) data conversion (if applicable); (2) analysis of variance components and power analysis; (3) production of confidence intervals for variance components; and (4) visualization of the confidence intervals.

### Data conversion

For data that were recorded at the replicate‐level, such as the number of offspring dead or alive for survival, these data should be converted to the individual‐level to not underestimate phenotypic variance and influence variance component estimates (Puurtinen et al. 2009 and example below). The buildBinary function can assign a binary number (i.e., “0” or “1”) to two columns containing the number of offspring and copy information by the number of times equal to the number of offspring. The final data set will have a number of rows matching the total number of offspring. The buildMulti function is similar and can assign multiple numbers to multiple columns.

### Analysis of variance components

#### Normal data

The observLmer function is used for the standard model (i.e., random effects for dam, sire, and dam by sire) on observed normal data (e.g., continuous data) with the Gaussian error structure. The advanced observLmer2 function can be used to model an additional random effect for position and/or block. The expert observLmer3 function can be used to model any additional fixed and random effects. The three functions have the option of estimating the parameters using maximum likelihood (ML) or restricted maximum likelihood (REML). ML estimates the parameters that maximize the likelihood of the observed data and has the advantage of using all the data and accounting for nonindependence (Lynch and Walsh 1998, p. 779; Bolker et al. 2009). On the other hand, ML has the disadvantage of assuming that all fixed effects are known without error, producing a downward bias in the estimation of the residual variance component. This bias can be large if there are lots of fixed effects, especially if sample sizes are small. REML has the advantage of not assuming the fixed effects are known and averages over the uncertainty, so there can be less bias in the estimation of the residual variance component. However, REML only maximizes a portion of the likelihood to estimate the effect parameters, but is the preferred method for analyzing large data sets with complex structure.

All three functions extract the dam, sire, dam by sire, and residual variance component. The observLmer2 and observLmer3 functions extract any additional random effects and/or fixed effects variance components. The component for fixed effects is extracted as a single group by multiplying the design matrix of the fixed effects with a vector of fixed effects estimates (Snijders and Bosker 1999; Nakagawa and Schielzeth 2013). The total variance is calculated and each component is presented in its raw and percentage values.

Two separate methods were used for determining the significance of the random effects and fixed effects. For each random effect, we used the recommended likelihood ratio test (LRT) comparing the full model to a reduced model without the single random effect (Bolker et al. 2009), and the function presents the *Χ* ^2^ statistic, difference in Akaike information criterion (ΔAIC) value, difference in Bayesian information criterion (ΔBIC) value, and *P*‐value (degree of freedom is always 1). There are options for ML and REML for the LRT. For determining the significance of each fixed effect, because LRT is not generally recommended for fixed effects and there are issues calculating the denominator degrees of freedom, we used a parametric bootstrap method (Bolker et al. 2009). Specifically, we integrated the parametric bootstrap mixed function of the afex package (Singmann et al. 2015) into the observLmer3 function, which produces a base distribution of likelihood *Χ* ^2^ statistics using ML, that is, then used for providing a *P*‐value for the observed *Χ* ^2^ statistic. Because LRT with ML is still used as an approximation of the significance of fixed effects (Pinheiro and Bates 2000), we also provide the LRT *Χ* ^2^ statistic, ΔAIC, ΔBIC, and *P*‐value.

The powerLmer, powerLmer2, and powerLmer3 functions are used for the power analyses of the variance components using normal data. Power values are calculated by stochastically simulating data for a number of iterations and then calculating the proportion of *P*‐values less than *α* (e.g., 0.05) for each component (Bolker 2008). *P*‐values for the random and fixed effects are calculated using REML or ML or parametric bootstrap as described above. Simulated data are specified by inputs for known variance component values and the sample sizes.

#### Nonnormal data

Equivalents to the lmer functions are available for observed nonnormal data (e.g., binary, proportion, and count data), specifically the observGlmer, observGlmer2, and observGlmer3 functions. There are also three functions for nonnormal data power analysis, that is, powerGlmer, powerGlmer2, and powerGlmer3. The three observed functions estimate parameters using Laplace approximation because there were more advantages relative to penalized quasi‐likelihood and Gauss–Hermite quadrature parameter estimation methods; that is, penalized quasi‐likelihood is not recommended for count responses with means less than five and binary responses with less than five successes per group. Gauss–Hermite quadrature is not recommended for more than two or three random effects because of the rapidly declining analytical speed with the increasing number of random effects. Because Laplace approximation is a true likelihood method (Bolker et al. 2009), the likelihood ratio tests use ML. The three glmer observed functions extract the variance components and perform the same calculations and significance tests as their lmer equivalents.

Binomial and Poisson error structures with four links are supported by the three glmer functions because the residual variance component of these error structures and links are specified by Nakagawa and Schielzeth (2010, 2013). Specifically, the residual variance component for binomial errors with the logit link is *π* ^2^/3; binomial errors with the probit link is 1; Poisson errors with the log link is ln(1/exp(*β*_0_) + 1), where *β*_0_ is the intercept value from the model without any fixed effects and containing only the random effects; and Poisson errors with the square‐root link is 0.25. We have also included an option to account for overdispersion of proportion data (i.e., quasi‐binomial) and count data (i.e., quasi‐Poisson); there is no overdispersion with binary data (Crawley 2005). Specifically, an additional observation‐level random effect is added to the model to account, and also act as a test, for overdispersion (Atkins et al. 2013).

### Bootstrap confidence intervals for variance components

Confidence intervals for the additive genetic, nonadditive genetic, and maternal variance components can be produced using the bootstrap‐*t* resampling method described by Efron and Tibshirani (1993, p. 160–162). Observations are resampled with replacement until the original sample size is reproduced. The resampled data are then used in the model, and the additive genetic, nonadditive genetic, and maternal variance components are extracted. The process is repeated for a number of iterations, typically 1000 times, to produce a distribution for each component. The confidence interval lower and upper limits and median are extracted from the distribution using R's generic quantile function. The resampRepli function is used to bootstrap resample observations grouped by replicate identities within family identities for a specified number of iterations to create the resampled data set. Because of the large file sizes that can be produced, the resampling of each family is saved separately as a common separated (.csv) file in the working directory, and these files are merged to create the final bootstrap resampled data set. A similar resampFamily function is able to resample observations grouped by family identities only.

Next, equivalents to the observed data lmer and glmer functions are available for the final bootstrap resampled data set, that is, resampLmer, resampLmer2, and resampLmer3 and resampGlmer, resampGlmer2, and resampGlmer3. The functions provide a data frame with columns containing the raw variance components for dam, sire, dam by sire, residual, total, additive genetic, nonadditive genetic, and maternal. Additional variance components for each additional random effect and additional fixed effects as one group can also be provided in columns. The number of rows in the data frame matches the number of iterations in the resampled data set, and each row represents a model number.

The ciMANA function is used to extract the bootstrap‐*t* confidence intervals and median for the additive genetic, nonadditive genetic, and maternal values from the data frame of models. Similarly, advanced ciMANA2 and expert ciMANA3 can be used to extract the confidence intervals and median from additional columns in the data frame of models. The confidence level is specified as a percentage (1 − *α*). The raw values are presented and are converted to a percentage of the total variance for each model.

Another advantage of the bootstrap‐*t* method is the statistical comparisons of additive genetic, nonadditive genetic, and maternal variance components between pairwise groups, such as populations (Houde et al. 2013, 2015). Using the resampled data sets, for one group the proportion of comparisons (i.e., variance components) that are either larger or smaller than the other group is calculated. The proportion serves as a one‐tailed *P*‐value testing for differences between groups. For example, using two groups and 1000 iterations for each group, the difference between groups is calculated for each paired iteration number. If there are less than 50 instances (*α *= 5% or 50 of 1000 iterations) with a difference less than zero or less than 50 instances with a difference greater than zero, there is a significant difference between groups.

The bootstrap‐*t* method may produce medians that are largely different from the observed values (Efron and Tibshirani 1993). The BCa method described by Efron and Tibshirani (1993, p. 184–188) can be used for the correction of bootstrap‐*t* confidence intervals. We have integrated into the bootstrap‐*t* confidence interval functions, that is, ciMANA, ciMANA2, ciMANA3, inputs for bias correction using the raw observed variance component values and acceleration correction using the delete‐one observation jackknife data set (e.g., JackLmer, see below).

### Jackknife confidence intervals for variance components

Confidence intervals for the additive genetic, nonadditive genetic, and maternal variance components can also be produced using the jackknife resampling method described by Efron and Tibshirani (1993, p. 141–145). The mean and the standard error of pseudo‐values for each variance component are calculated. The standard error is then used with Student's *t‐*distribution to provide the lower and upper limits for the confidence interval. Because the delete‐one observation jackknife resampling may be computationally intensive for large data sets, the functions have the option of delete‐*d* observation jackknife resampling. We used *M* degrees of freedom for producing the confidence interval (Martin et al. 2004): *M *= *N*/*d*, where *N* is the total number of observations and *d* is the number of deleted observations. Large values of *M*, such as 1000, can translate to the delete‐*d* jackknife resampling method approaching bootstrap resampling expectations (Efron and Tibshirani 1993, p. 149).

Equivalents to the observed data lmer and glmer functions are available for jackknife resampling, that is, JackLmer, JackLmer2, and JackLmer3 and JackGlmer, JackGlmer2, and JackGlmer3. The default is delete‐one jackknife resampling. For the option of delete‐*d* jackknife resampling, the rows of the observed data frame are shuffled and a block of observations of size *d* is deleted sequentially. The functions provide a data frame with columns containing the raw variance components for dam, sire, dam by sire, residual, total, additive genetic, nonadditive genetic, and maternal. Additional variance components for each addition random effect and additional fixed effects as one group can also be provided in columns. The number of rows in the data frame matches the total number of observations (*N*) for delete‐one jackknife resampling or *M* groups for delete‐*d* jackknife resampling to the lowest integer. Each row represents a deleted single observation or deleted‐*d* observations group.

The ciJack function is used to extract the jackknife confidence intervals and pseudo‐value means of the additive genetic, nonadditive genetic, and maternal variance components from the jackknife data frame. Similarly, advanced ciJack2 and expert ciJack3 can be used to extract the confidence intervals and pseudo‐value means from additional columns in the data frame of models. The functions have inputs for the raw observed variance component values to calculate the pseudo‐values. The confidence level is specified as a percentage (1 − *α*). The raw values are presented and are converted to a percentage of the total variance for each row of the jackknife data frame.

### Visualization of the confidence intervals

The barMANA and boxMANA functions are simple plotting functions for the confidence intervals or all values from the bootstrap and jackknife data frames. The barMANA function produces bar graphs with the median or pseudo‐value mean as the top of the shaded bar and error bars covering the range of the confidence interval for each of the additive genetic, nonadditive genetic, and maternal values of a phenotypic trait, as well as producing a simple legend. The boxMANA function produces boxplots using all values for three components. In addition, the functions can plot several graphs grouped by labels to visualize several phenotypic traits. Within the functions, there are simple plot modifications available, such as changing the range of the *y*‐axis and the length of the error bars.

## Worked Example: Chinook Salmon Survival to Hatching

An 11 × 11 factorial mating design was used to produce Chinook salmon offspring, that is, crossing 11 dams with 11 sires in all possible pairwise combinations (additional details of methods and original two‐way ANOVA are described in Pitcher and Neff (2007)). There were two replicates for each of the 121 families, each containing 150 eggs or individuals. Each family replicate was haphazardly placed into an incubation cell (*n *=* *16) within a tray (*n *=* *16). A subsample of 10 eggs per dam was measured for egg diameter (nearest 0.1 mm) using digital calipers; mean egg diameter per dam was used in the analyses. The number of individuals (i.e., counts) that died before hatching or survived to hatching was collected for each of the two replicates per family. In this example, we go through the entire workflow for survival as a binary variable and we also demonstrate how using the original replicate‐level data can influence variance component estimates. Statistical significance is set at *α *= 0.05. A simulated data example for survival and another worked example for a continuous variable (i.e., length at hatch) are provided in the supplementary information.

### Step 1: Analysis of variance components

Because the data were recorded at the replicate‐level, we will convert the data to the individual‐level using the buildBinary function. The input contains the original data (i.e., chinook_survival) with the column numbers (1–6 and 9) to copy corresponding to family, replicate, dam, sire, tray, cell, and egg size. The input also contains two column names for the number of individuals to be assigned a “1” value and a “0” value in quotations, that is, “alive” and “dead.” The output is a data frame that contains a new column named “status.”
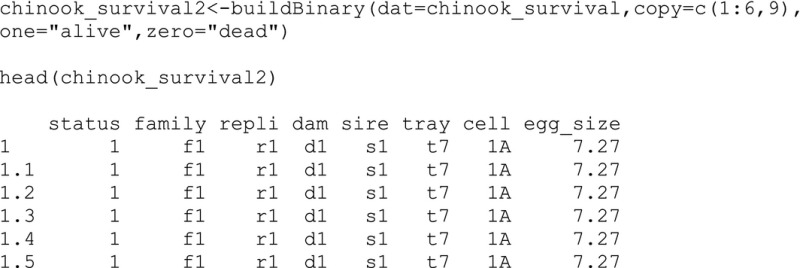


Because we are interested in examining whether there is a relationship between survival and egg size and accounting for two potential position effects (i.e., tray and cell) that may be nuisance sources of environmental variance contributing to the phenotype, the initial model (an object named “survival_mod1”) includes one fixed effect for the mean egg diameter of each dam (i.e., egg size), one random effect for tray, and one random effect for cell. We will use the observGlmer3 function because it can handle fixed effects. The input contains the observed data frame (i.e., chinook_survival2), and the column names contain the dam, sire, and response in quotations. The input also contains the family (i.e., error structure) and link using the R format. Because the individual‐level survival data are binary, for “fam_link” we use “binomial(logit).” Internally, the function has the dam, sire, and dam by sire as random effects, so the input also contains the “remain” fixed and random effects for the model in quotations using the lme4 package formula format (Bates et al. 2015). 
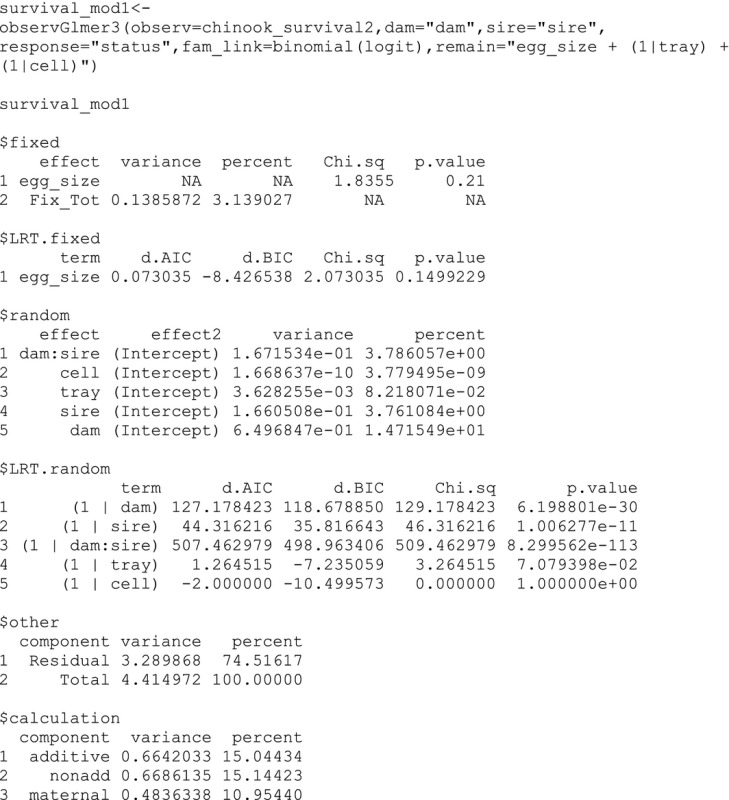


The output object contains significant random effects for dam (14.7% of the phenotypic variance), sire (3.8%), and dam by sire (3.8%). Additive genetic, nonadditive genetic, and maternal variance explain similar amounts of the phenotypic variance (15.0%, 15.1%, and 11.0%). With the addition of the residual component, the components can sum to more than 100% if there is epistasis (Neff and Pitcher 2005). None of the fixed effects (i.e., egg size) or random effects for position (i.e., tray and cell) were significant, so we can reduce the number of effects and use the observGlmer function to make a new reduced model, which has results similar to the full model (see supplementary information). Below, we evaluate the power of this new model using the powerGlmer function with 300 offspring per family and 500 simulations. The power values were larger than 0.8% or 80% for the dam, sire, and dam by sire variance components. 
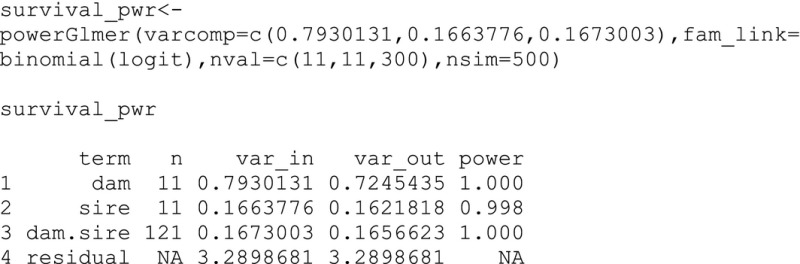


### Step 2: Production of confidence intervals for variance components

To produce bootstrap‐*t* confidence intervals for the additive genetic, nonadditive genetic, and maternal variance components, first the observed data for every replicate within family are resampled with replacement for a given number of iterations using the resampRepli function. The input contains the observed data frame (i.e., chinook_survival2) and the column numbers to copy as a vector, so we include the column numbers for response (i.e., status, 1), dam (4), and sire (5). The input also contains the column names for the family column and replicate column in quotations. The final resampled data set will be located in the working directory. 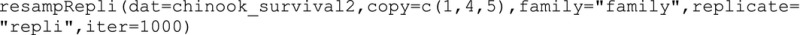


Second, we apply a common model to each iteration of the resampled data set using the resampGlmer function. The input contains the final resampled data set (i.e., chinook_resampS), the column names for dam, sire, and the response in quotations, and the starting model iteration and ending model iteration. Each row of the output represents a model iteration number. 
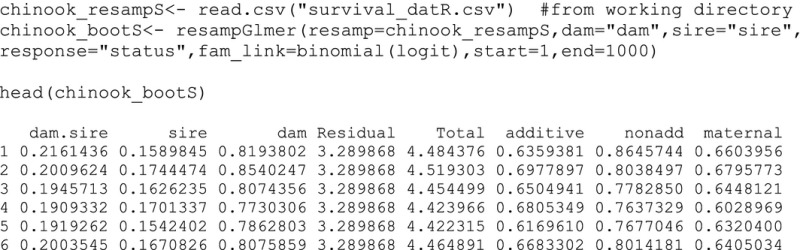


Third, we extract the bootstrap‐*t* confidence intervals and median from the variance component data frame using the ciMANA function. The input is the variance component data frame (i.e., chinook_bootS). We used the default confidence level of 95% because our *α* is 0.05 for statistical significance and the default percentage rounding off to one decimal place. Internally, all variance components within a row are converted to percentages of the total variance value of that row. The output is presented as raw values and the percentages. 
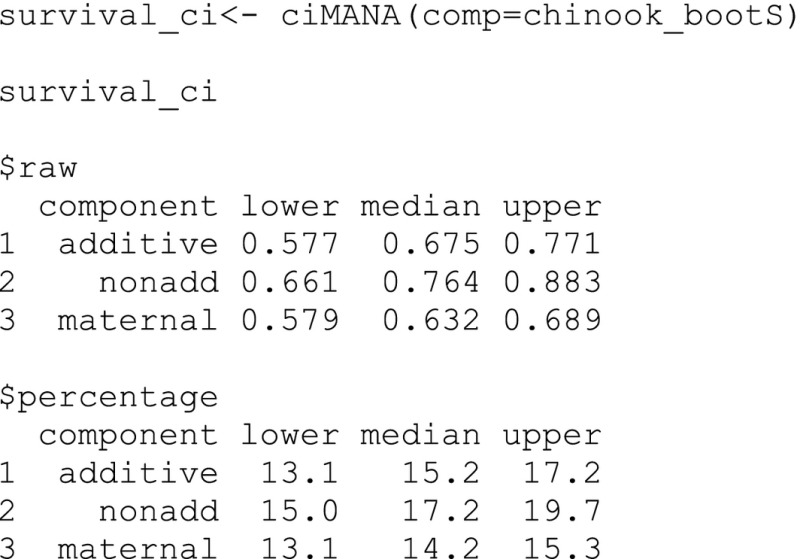


### Step 3: Visualization of the confidence intervals

**Figure 2:**
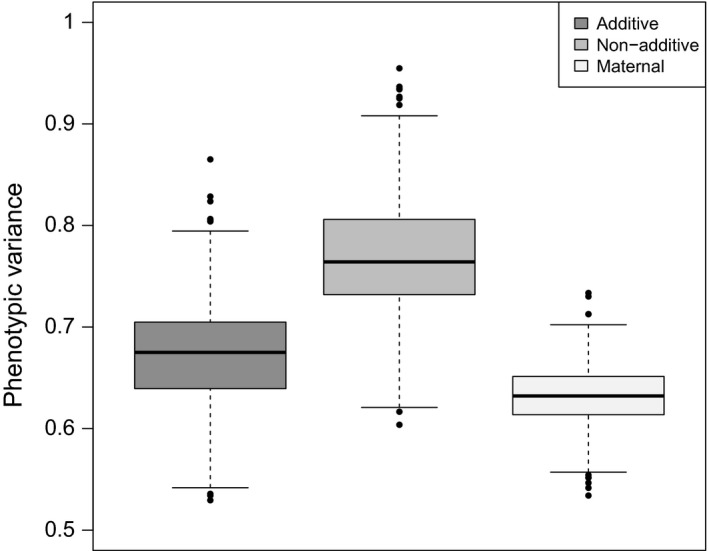
Boxplots of the additive genetic, nonadditive genetic, and maternal effects underlying the phenotypic variance of the survival to hatching for Chinook salmon (*Oncorhynchus tshawytscha*). The lower and upper ends of each box represent the 25th and 75th quartiles, respectively. Medians are represented by the bold bar in each box. Outliers are represented by dots that are 1.5 times the interquantile range. Code is as follows: box MANA (comp=chinook_bootS, type=“raw”, yunit=0.1, ymin=0.5, ymax=1, cex_ylab=1.3).

A simple plot of the additive genetic, nonadditive genetic, and maternal raw variance component values is produced using the boxMANA function (Fig. 2). The input contains the variance component data frame (i.e., chinook_bootS) and opens an R graphics device window. After looking at the initial plot (not shown), we included additional input parameters for the unit increment of the *y*‐axis, minimum and maximum values of the *y*‐axis, and the size of the label on the *y*‐axis. The plot shows that additive genetic, nonadditive genetic, and maternal effects are contributing to the phenotypic variance of survival to hatching for the Chinook salmon 11 × 11 factorial mating.

### Analysis of variance components: individual‐level versus replicate‐level data

To demonstrate how using replicate‐level survival data can influence variance components, we used the original replicate‐level proportion data (i.e., proportion of individuals alive to hatch) and compared it to the individual‐level binary data using the same “fam_link.” We previously tested for overdispersion of the proportion data using the option in the input of observGlmer function, which was nonsignificant, so the overdispersion parameter was removed (see supplementary information). 
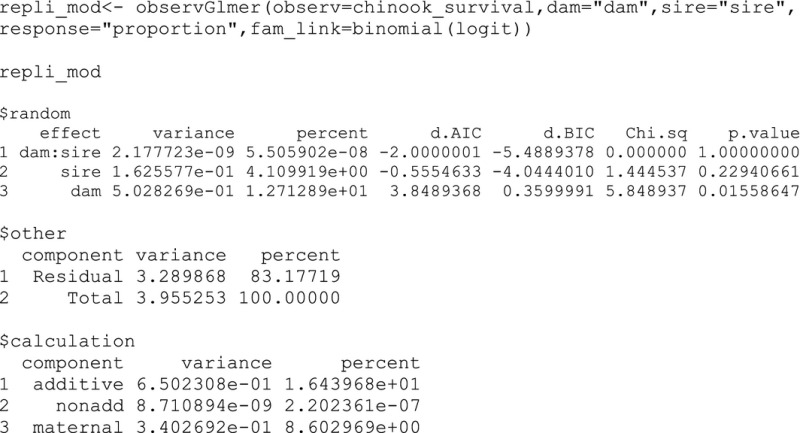


The analysis with the proportion data displays slightly higher additive genetic variance by 1.3%, whereas the nonadditive genetic variance largely decreased and is now close to 0% and the maternal variance decreased by 5.6%, relative to the analysis with binary data. A similar effect is observed using the family means of length at hatch per replicate; there was a large decrease in nonadditive genetic variance but maternal variance increased in this case (see supplementary information). For both the survival and length data, there was a decrease in the phenotypic (total) variance using means of replicates as suggested by Puurtinen et al. (2009). However, Puurtinen et al. (2009) also suggested an increase in genetic variance, but our analyses suggest that changes in additive genetic variance may be minor compared to the large decreases in nonadditive genetic variance and apparently variable changes in maternal variance for certain traits.

## Conclusion

We aimed to produce an analytical tool for mixed‐effects models in R to be used with full factorial mating designs. Mixed‐effects models are appropriate for unbalanced designs and do not produce negative components, relative to the traditional two‐way ANOVA (Lynch and Walsh 1998). The fullfact package contains functions to calculate additive genetic, nonadditive genetic, and maternal variance components that explain the phenotype, as well as providing significance values, power values, and confidence intervals for these components. This package also contains various functions that build on the analysis of full factorial mating designs by providing: (1) more accurate estimates of phenotypic variance; (2) incorporating the residual variance components for nonnormal error structures (e.g., binary, proportion, and/or count data types); and (3) tests of the significance of any additional variables (i.e., additional random and fixed effects). The fullfact package will ultimately facilitate and enhance the analyses of full factorial mating designs in R.

## Conflict of Interest

None declared.

## Supporting information


**Data S1.** Additional models from Chinook salmon survival to hatching worked example described in main text.
**Data S2.** Simulated data example of survival.
**Data S3.** Worked example for Chinook salmon length at hatch.
