# Supplementary material for: fullfact: an R package for the analysis of genetic and maternal variance components from full factorial mating designs
Source: Ecol Evol. 2016 Feb 14;6(6):1656–65. doi: 10.1002/ece3.1943 (PMC4752957; doi:10.1002/ece3.1943)
Supplement: Supplementary file 5 [file ECE3-6-1656-s005.pdf]

Package ‘fullfact’

December 1, 2015

Version 1.0

Date 2015-12-01

Title Full Factorial Breeding Analysis

Author Aimee Lee Houde [aut, cre], Trevor Pitcher [aut]

Maintainer Aimee Lee Houde <aimee.lee.houde@gmail.com>

Depends R (>= 3.2.2), lme4, afex

Description Package for the analysis of full factorial breeding designs.

License GPL (>=2)

URL http://www.r-project.org

BugReports Aimee Lee Houde <aimee.lee.houde@gmail.com>

NeedsCompilation no

R topics documented:

fullfact-package 2
barMANA 5
boxMANA 6
buildBinary 8
buildMulti 9
chinook_bootL 10
chinook_bootS 11
chinook_jackL 12
chinook_length 13
chinook_resampL 14
chinook_resampS 15
chinook_survival 16
ciJack 17
ciJack2 19
ciJack3 20
ciMANA 21
ciMANA2 22
ciMANA3 24
JackGlmer 25
JackGlmer2 27
JackGlmer3 29
JackLmer 31

JackLmer2 33
JackLmer3 35
observGlmer 37
observGlmer2 38
observGlmer3 40
observLmer 42
observLmer2 43
observLmer3 44
powerGlmer 46
powerGlmer2 47
powerGlmer3 49
powerLmer 51
powerLmer2 53
powerLmer3 54
resampFamily 56
resampGlmer 57
resampGlmer2 59
resampGlmer3 60
resampLmer 62
resampLmer2 64
resampLmer3 65
resampRepli 67

Index **68**

`fullfact-package` *Full Factorial Breeding Analysis*

Description

Analysis of full factorial breeding designs. The package incorporates mixed-effects models suited for unbalanced experimental designs and non-normal data types. Extracts variance components and significance values for random and fixed effects. Calculates the amount of additive genetic, non-additive genetic, and maternal variance explaining phenotypic traits. Produces confidence values using a bootstrap resampling routine and can apply bias and acceleration correction. Also includes an option for producing jackknife resampling confidence intervals. Calculates the power values of random and fixed effects.

Details

The DESCRIPTION file:

Package:	fullfact
Version:	1.0
Date:	2015-12-01
Title:	Full Factorial Breeding Analysis
Author:	Aimee Lee Houde [aut, cre], Trevor Pitcher [aut]
Maintainer:	Aimee Lee Houde <aimee.lee.houde@gmail.com>
Depends:	R (>= 3.2.2), lme4, afex
Description:	Package for the analysis of full factorial breeding designs.
License:	GPL (>=2)
URL:	http://www.r-project.org

BugReports: Aimee Lee Houde <aimee.lee.houde@gmail.com>

Index of help topics:

```
JackGlmer               Jackknife components for non-normal data
JackGlmer2              Jackknife components for non-normal data 2
JackGlmer3              Jackknife components for non-normal data 3
JackLmer                Jackknife components for normal data
JackLmer2               Jackknife components for normal data 2
JackLmer3               Jackknife components for normal data 3
barMANA                 Bargraph of confidence intervals
boxMANA                 Boxplot of resampled results
buildBinary             Convert to a binary data frame
buildMulti              Convert to a multinomial frame
chinook_bootL           Chinook salmon length, bootstrap calculations
chinook_bootS           Chinook salmon survival, bootstrap data
chinook_jackL           Chinook salmon length, jackknife data
chinook_length          Chinook salmon length, raw data
chinook_resampL         Chinook salmon length, bootstrap resampled
chinook_resampS         Chinook salmon survival, bootstrap resampled
chinook_survival        Chinook salmon survival, raw data
ciJack                  Jackknife confidence intervals
ciJack2                 Jackknife confidence intervals 2
ciJack3                 Jackknife confidence intervals 3
ciMANA                  Bootstrap confidence intervals
ciMANA2                 Bootstrap confidence intervals 2
ciMANA3                 Bootstrap confidence intervals 3
fullfact-package        Full Factorial Breeding Analysis
observGlmer             Variance components for non-normal data
observGlmer2            Variance components for non-normal data 2
observGlmer3            Variance components for non-normal data 3
observLmer              Variance components for normal data
observLmer2             Variance components for normal data 2
observLmer3             Variance components for normal data 3
powerGlmer              Power analysis for non-normal data
powerGlmer2             Power analysis for non-normal data 2
powerGlmer3             Power analysis for non-normal data 3
powerLmer               Power analysis for normal data
powerLmer2              Power analysis for normal data 2
powerLmer3              Power analysis for normal data 3
resampFamily            Bootstrap resample within families
resampGlmer             Bootstrap components for non-normal data
resampGlmer2            Bootstrap components for non-normal data 2
resampGlmer3            Bootstrap components for non-normal data 3
resampLmer              Bootstrap components for normal data
resampLmer2             Bootstrap components for normal data 2
resampLmer3             Bootstrap components for normal data 3
resampRepli             Bootstrap resample within replicates
```

Author(s)

Aimee Lee Houde [aut, cre], Trevor Pitcher [aut]

Maintainer: Aimee Lee Houde <aimee.lee.houde@gmail.com>

References

Traditional full factorial breeding design analysis:

Lynch M, Walsh B. 1998. Genetics and Analysis of Quantitative Traits. Sinauer Associates, Massachusetts.

Residual variance component values for generalized linear mixed-effects models:

Nakagawa S, Schielzeth H. 2010. Repeatability for Gaussian and non-Gaussian data: a practical guide for biologists. Biological Reviews 85(4): 935-956. DOI: 10.1111/j.1469-185X.2010.00141.x

Fixed effect variance component values for mixed-effects models:

Nakagawa S, Schielzeth H. 2013. A general and simple method for obtaining R2 from generalized linear mixed-effects models. Methods in Ecology and Evolution 4(2): 133-142. DOI: 10.1111/j.2041-210x.2012.00261.x

Confidence intervals (bootstrap resampling, bias and acceleration correction, jackknife resampling):

Efron B, Tibshirani R. 1993. An introduction to the Bootstrap. Chapman and Hall, New York.

Martin, H., Westad, F. & Martens, H. (2004). Imporved Jackknife Variance Estimates of Bilinear Model Parameters. COMPSTAT 2004 – Proceedings in Computational Statistics 16th Symposium Held in Prague, Czech Republic, 2004 (ed J. Antoch), pp. 261-275. Physica-Verlag HD, Heidelberg.

Data sources:

Pitcher TE, Neff BD. 2007. Genetic quality and offspring performance in Chinook salmon: implications for supportive breeding. Conservation Genetics 8(3):607-616. DOI: 10.1007/s10592-006-9204-z

Examples

```
data(chinook_length) #Chinook salmon offspring length

## Standard additive genetic, non-additive genetic, and maternal variance analysis

length_mod1<- observLmer(observ=chinook_length,dam="dam",sire="sire",response="length")
length_mod1

## Confidence intervals

##Bootstrap resampling of data: replicates within family
#resampRepli(dat=chinook_length,copy=c(3:8),family="family",replicate="repli",iter=1000)
resampRepli(dat=chinook_length,copy=c(3:8),family="family",replicate="repli",iter=5)
#example of 5 iterations
#saves the files in working directory: one for each replicate and
#one final (combined) file "resamp_datR.csv"

##Import file
#length_datR<- read.csv("resamp_datR.csv")
data(chinook_resampL) #same as length_datR, 5 iterations

##Models for the resampled data: standard analysis
#length_rcomp<- resampLmer(resamp=length_datR,dam="dam",sire="sire",response="length",
#start=1,end=1000)
length_rcomp<- resampLmer(resamp=chinook_resampL,dam="dam",sire="sire",response="length",
start=1,end=5) #example of 5 models
```

```
## 1. Uncorrected Bootstrap 95% confidence interval

#ciMANA(comp=length_rcomp)
data(chinook_bootL)  #similar to length_rcomp, but 1,000 models
ciMANA(comp=chinook_bootL)

## 2. Bias and accelerated corrected Bootstrap 95% confidence interval

##Jackknife resampling of data, delete-one: for acceleration estimate
#length_jack<- JackLmer(observ=chinook_length,dam="dam",sire="sire",
#response="length")
length_jack<- JackLmer(observ=chinook_length,dam="dam",sire="sire",
response="length",first=10) #example of first 10 observations

#ciMANA(comp=length_rcomp,bias=c(0.0000000,0.7192253,0.2029684),accel=length_jack)
data(chinook_jackL)  #similar to length_jack, but all observations
ciMANA(comp=chinook_bootL,bias=c(0.0000000,0.7192253,0.2029684),accel=chinook_jackL)

##3. Jackknife 95% confidence interval

#ciJack(comp=length_jack,full=c(0.0000000,0.7192253,0.2029684,1.0404425))
ciJack(comp=chinook_jackL,full=c(0.0000000,0.7192253,0.2029684,1.0404425))
```

---

barMANA *Bargraph of confidence intervals*

Description

A simple bargraph function for confidence intervals of additive genetic, non-additive genetic, and maternal variance components. Also, plots the median for the bootstrap resampling method or mean of the pseudo-values for the jackknife resampling method.

Usage

```
barMANA(ci_dat, type = "perc", bar_len = 0.1, ymax = NULL, ymin = NULL, yunit = NULL,
leg = "topright", cex_ylab = 1, cex_yaxis = 1, cex_names = 1)
```

Arguments

ci_dat	Data frame of a confidence interval function.
type	Default is "perc" for percentage values of variance components. Other option is "raw" for raw values of variance components.
bar_len	Length of error bar in inches.
ymax	Maximum value of the y-axis.
ymin	Minimum value of the y-axis.
yunit	Unit increment of the y-axis.
leg	Position of the simple legend.
cex_ylab	Magnification of the y-axis label.
cex_yaxis	Magnification of the y-axis units.
cex_names	Optional magnification of trait labels.

Details

Plots a bargraph with the median or mean as the top of the shaded bar and error bars covering the range of the confidence interval. Uses an object produced by any of the bootstrap resampling CI functions, i.e. *ciMANA*, *ciMANA2*, and *ciMANA3* or jackknife resampling functions, i.e. *ciJack*, *ciJack2*, and *ciJack3*. The median is plotted for bootstrap resampling and the mean of pseudo-value for jackknife resampling. Produces a simple legend. The function can plot several bar graphs grouped by *label* to visualize several phenotypic traits.

Examples

```
##Import bootstrap resampling results
data(chinook_bootS) #Chinook salmon offspring survival
#Extract un-corrected confidence interval
survival_ci<- ciMANA(comp=chinook_bootS,trait="survival")
survival_ci

#Default plot
barMANA(ci_dat=survival_ci)
#Add plot modifications
barMANA(ci_dat=survival_ci,bar_len=0.3,yunit=5,ymax=20,cex_ylab=1.3)

##Import jackknife resampling results
data(chinook_jackL)  #Chinook salmon offspring length
#Extract jackknife confidence interval
length_ci<- ciJack2(comp=chinook_jackL,full=c(0.0000000,0.7192253,0.2029684,1.0404425,
0.1077423),position="tray",trait="length")
length_ci

#Default plot
barMANA(ci_dat=length_ci)
#Add plot modifications
barMANA(ci_dat=length_ci,bar_len=0.3,yunit=20,ymax=100,cex_ylab=1.3)

##Group survival and length together in the same plot
data(chinook_bootL) #Chinook salmon offspring length
length_ci2<- ciMANA2(comp=chinook_bootL,position="tray",trait="length")
length_ci2
#
comb_r<- rbind(survival_ci$raw,length_ci2$raw)
comb_p<- rbind(survival_ci$percentage,length_ci2$percentage)
comb_ci<- list(raw=comb_r,percentage=comb_p)

#Default plot
barMANA(ci_dat=comb_ci)
#Add plot modifications
barMANA(ci_dat=comb_ci,bar_len=0.3,yunit=20,ymax=100,cex_ylab=1.3,leg="topleft")
```

---

boxMANA *Boxplot of resampled results*

Description

A simple boxplot function for bootstrap and jackknife resampled results of additive genetic, non-additive genetic, and maternal variance components.

Usage

```
boxMANA(comp, type = "perc", ymax = NULL, ymin = NULL, yunit = NULL, leg = "topright",
cex_ylab = 1, cex_yaxis = 1, cex_names = 1)
```

Arguments

comp	Data frame of bootstrap or jackknife resampling results.
type	Default is "perc" for percentage values of variance components. Other option is "raw" for raw values of variance components.
ymax	Maximum value of the y-axis.
ymin	Minimum value of the y-axis.
yunit	Unit increment of the y-axis.
leg	Position of the simple legend.
cex_ylab	Magnification of the y-axis label.
cex_yaxis	Magnification of the y-axis units.
cex_names	Optional magnification of trait labels.

Details

Plots an R boxplot. Uses an object produced by any of the bootstrap resampling functions, i.e. *resampLmer*, *resampLmer2*, *resampLmer3*, *resampGlmer*, *resampGlmer2*, and *resampGlmer3*. Or any of the jackknife resampling functions, i.e. *JackLmer*, *JackLmer2*, *JackLmer3*, *JackGlmer*, *JackGlmer2*, and *JackGlmer3*. Produces a simple legend.

Examples

```
#Import bootstrap resampled data model results
data(chinook_bootL) #Chinook salmon offspring length

#Default plot
boxMANA(comp=chinook_bootL)
#Add plot modifications
boxMANA(comp=chinook_bootL,yunit=20,ymax=100,cex_ylab=1.3)

##Group length and survival together in the same plot
data(chinook_bootS) #Chinook salmon offspring survival
chinook_bootL$trait<- "length"
chinook_bootS$trait<- "survival"

colnames(chinook_bootL[,-2])
colnames(chinook_bootS)
#
comb_boot<- rbind(chinook_bootL[,-2],chinook_bootS) #remove 'tray'
comb_boot$trait<- as.factor(comb_boot$trait) #to form levels

#Default plot
boxMANA(comp=comb_boot)
#Add plot modifications
boxMANA(comp=comb_boot,yunit=20,ymax=100,cex_ylab=1.3)
```

`buildBinary` *Convert to a binary data frame*

Description

Assign a binary number (i.e. '0' or '1') to two columns containing the number of offspring. Copy information by the number of times equal to the number of offspring.

Usage

```
buildBinary(dat, copy, one, zero)
```

Arguments

`dat`	Data frame to convert.
`copy`	Column numbers to copy.
`one`	Column name of counts to assign a '1' value.
`zero`	Column name of counts to assign a '0' value.

Details

Replicate-level data should be converted to the individual-level to not underestimate phenotypic variance, which can influence genetic and maternal estimates (see Puurtinen et al. 2009).

Value

A converted data frame with a number of row matching the total number of individuals.

References

Puurtinen M, Ketola T, Kotiaho JS. 2009. The good-genes and compatible-genes benefits of mate choice. The American Naturalist 174(5): 741-752. DOI: 10.1086/606024

See Also

`buildMulti`

Examples

```
data(chinook_survival)
chinook_survival2<- buildBinary(dat=chinook_survival,copy=c(1:6,9),one="alive",zero="dead")
```

buildMulti *Convert to a multinomial frame*

Description

Assign multiple numbers to multiple columns containing the number of offspring. Copy information by the number of times equal to the number of offspring.

Usage

```
buildMulti(dat, copy, multi)
```

Arguments

`dat`	Data frame to convert.
`copy`	Column numbers to copy.
`multi`	A list containing the numbers to assign and matching column names. E.g. list(c(2,0,1),c("two","zero","one").)

Details

Replicate-level data should be converted to the individual-level to not underestimate phenotypic variance, which can influence genetic and maternal estimates (see Puurtinen et al. 2009).

Value

A converted data frame with a number of row matching the total number of individuals.

References

Puurtinen M, Ketola T, Kotiaho JS. 2009. The good-genes and compatible-genes benefits of mate choice. The American Naturalist 174(5): 741-752. DOI: 10.1086/606024

See Also

`buildBinary`

Examples

```
data(chinook_survival)
chinook_survival$total<- chinook_survival$alive + chinook_survival$dead #create total column
chinook_survival3<- buildMulti(dat=chinook_survival,copy=c(1:6,9),multi=list(c(2,1,0),
c("total","alive","dead")))
```

chinook_bootL *Chinook salmon length, bootstrap calculations*

Description

Bootstrap resampled Chinook salmon fork length (mm) at hatch with the amount of additive genetic, non-additive genetic, and maternal variance calculations.

Usage

```
data("chinook_bootL")
```

Format

A data frame with 1000 observations on the following 9 variables.

- `dam.sire`, a numeric vector.
- `tray`, a numeric vector.
- `sire`, a numeric vector.
- `dam`, a numeric vector.
- `Residual`, a numeric vector.
- `Total`, a numeric vector.
- `additive`, a numeric vector.
- `maternal`, a numeric vector.
- `nonadd`, a numeric vector.

Details

Also includes the calculations for the amount of variance explained by position (tray), dam by sire, sire, dam, residual,and total.

Source

http://link.springer.com.proxy1.lib.uwo.ca/article/10.1007

References

Pitcher TE, Neff BD. 2007. Genetic quality and offspring performance in Chinook salmon: implications for supportive breeding. Conservation Genetics 8(3):607-616. DOI: 10.1007/s10592-006-9204-z

Examples

```
data(chinook_bootL)
## Extract bootstrap confidence interval
ciMANA(comp=chinook_bootL)
```

`chinook_bootS` *Chinook salmon survival, bootstrap data*

Description

Bootstrap resampled Chinook salmon binary survival to hatch (1 is alive, 0 is dead) with the amount of additive genetic, non-additive genetic, and maternal variance calculations.

Usage

```
data("chinook_bootS")
```

Format

A data frame with 1000 observations on the following 8 variables.

`dam.sire`, a numeric vector.

`sire`, a numeric vector.

`dam`, a numeric vector.

`Residual`, a numeric vector.

`Total`, a numeric vector.

`additive`, a numeric vector.

`maternal`, a numeric vector.

`nonadd`, a numeric vector.

Details

Also includes the calculations for the amount of variance explained by dam by sire, sire, dam, residual, and total.

Source

http://link.springer.com.proxy1.lib.uwo.ca/article/10.1007

References

Pitcher TE, Neff BD. 2007. Genetic quality and offspring performance in Chinook salmon: implications for supportive breeding. Conservation Genetics 8(3):607-616. DOI: 10.1007/s10592-006-9204-z

Examples

```
data(chinook_bootS)
## Extract bootstrap confidence interval
ciMANA(comp=chinook_bootS)
```

chinook_jackL *Chinook salmon length, jackknife data*

Description

Jackknife resampled Chinook salmon fork length (mm) at hatch with the amount of additive genetic, non-additive genetic, and maternal variance calculations. Jackknife resampling was leave-out-one.

Usage

```
data("chinook_jackL")
```

Format

A data frame with 1210 observations on the following 9 variables.

`dam.sire`, a numeric vector.

`tray`, a numeric vector.

`sire`, a numeric vector.

`dam`, a numeric vector.

`Residual`, a numeric vector.

`Total`, a numeric vector.

`additive`, a numeric vector.

`nonadd`, a numeric vector.

`maternal`, a numeric vector.

Details

Also includes the calculations for the amount of variance explained by position (tray), dam by sire, sire, dam, residual, and total.

Source

http://link.springer.com.proxy1.lib.uwo.ca/article/10.1007

References

Pitcher TE, Neff BD. 2007. Genetic quality and offspring performance in Chinook salmon: implications for supportive breeding. Conservation Genetics 8(3):607-616. DOI: 10.1007/s10592-006-9204-z

Examples

```
data(chinook_jackL)
## Extract jackknifed confidence interval
ciJack(comp=chinook_jackL,full=c(0.0000000,0.7192253,0.2029684,1.0404425))
```

chinook_length *Chinook salmon length, raw data*

Description

Raw Chinook salmon fork length (mm) at hatch for offspring produced using an 11 x 11 full factorial breeding design.

Usage

```
data("chinook_length")
```

Format

A data frame with 1210 observations on the following 8 variables.

`family`, a factor with levels: `f1 f10 f100 f101 f102 f103 f104 f105 f106 f107 f108 f109 f11 f110 f111 f112 f113 f114 f115 f116 f117 f118 f119 f12 f120 f121 f13 f14 f15 f16 f17 f18 f19 f2 f20 f21 f22 f23 f24 f25 f26 f27 f28 f29 f3 f30 f31 f32 f33 f34 f35 f36 f37 f38 f39 f4 f40 f41 f42 f43 f44 f45 f46 f47 f48 f49 f5 f50 f51 f52 f53 f54 f55 f56 f57 f58 f59 f6 f60 f61 f62 f63 f64 f65 f66 f67 f68 f69 f7 f70 f71 f72 f73 f74 f75 f76 f77 f78 f79 f8 f80 f81 f82 f83 f84 f85 f86 f87 f88 f89 f9 f90 f91 f92 f93 f94 f95 f96 f97 f98 f99`

`repli`, a factor with levels: `r1 r2`

`dam`, a factor with levels: `d1 d10 d11 d2 d3 d4 d5 d6 d7 d8 d9`

`sire`, a factor with levels: `s1 s10 s11 s2 s3 s4 s5 s6 s7 s8 s9`

`tray`, a factor with levels: `t1 t10 t11 t12 t13 t14 t15 t16 t2 t3 t4 t5 t6 t7 t8 t9`

`cell`, a factor with levels: `1A 1B 1C 1D 2A 2B 2C 2D 3A 3B 3C 3D 4A 4B 4C 4D`

`length`, a numeric vector.

`egg_size`, a numeric vector.

Details

Also includes family identity, family replicate, incubator position (tray and cell), and average female egg size (mm) information.

Source

http://link.springer.com.proxy1.lib.uwo.ca/article/10.1007

References

Pitcher TE, Neff BD. 2007. Genetic quality and offspring performance in Chinook salmon: implications for supportive breeding. Conservation Genetics 8(3):607-616. DOI: 10.1007/s10592-006-9204-z

Examples

```
data(chinook_length)
## Standard additive genetic, non-additive genetic, and maternal variance analysis
length_mod1<- observLmer(observ=chinook_length,dam="dam",sire="sire",response="length")
length_mod1
```

chinook_resampL *Chinook salmon length, bootstrap resampled*

Description

Bootstrap resampled Chinook salmon fork length (mm) at hatch. Number of iterations was 5.

Usage

```
data("chinook_resampL")
```

Format

A data frame with 1210 observations on the following 30 variables.

dam1, a numeric vector
sire1, a numeric vector
tray1, a numeric vector
cell1, a numeric vector
length1, a numeric vector
egg_size1, a numeric vector
dam2, a numeric vector
sire2, a numeric vector
tray2, a numeric vector
cell2, a numeric vector
length2, a numeric vector
egg_size2, a numeric vector
dam3, a numeric vector
sire3, a numeric vector
tray3, a numeric vector
cell3, a numeric vector
length3, a numeric vector
egg_size3, a numeric vector
dam4, a numeric vector
sire4, a numeric vector
tray4, a numeric vector
cell4, a numeric vector
length4, a numeric vector
egg_size4, a numeric vector
dam5, a numeric vector
sire5, a numeric vector
tray5, a numeric vector
cell5, a numeric vector
length5, a numeric vector
egg_size5, a numeric vector

Source

Pitcher TE, Neff BD. 2007. Genetic quality and offspring performance in Chinook salmon: implications for supportive breeding. Conservation Genetics 8(3):607-616. DOI: 10.1007/s10592-006-9204-z

Examples

```
data(chinook_resampL)
#the five models
length_rcomp1<- resampLmer(resamp=chinook_resampL,dam="dam",sire="sire",response="length",
start=1,end=5)  #full analysis should use 1,000 models
```

`chinook_resampS` *Chinook salmon survival, bootstrap resampled*

Description

Bootstrap resampled Chinook salmon binary survival to hatch (1 is alive, 0 is dead). Number of iterations was 5.

Usage

```
data("chinook_resampS")
```

Format

A data frame with 36300 observations on the following 30 variables.

`status1`, a numeric vector

`dam1`, a numeric vector

`sire1`, a numeric vector

`tray1`, a numeric vector

`cell1`, a numeric vector

`egg_size1`, a numeric vector

`status2`, a numeric vector

`dam2`, a numeric vector

`sire2`, a numeric vector

`tray2`, a numeric vector

`cell2`, a numeric vector

`egg_size2`, a numeric vector

`status3`, a numeric vector

`dam3`, a numeric vector

`sire3`, a numeric vector

`tray3`, a numeric vector

`cell3`, a numeric vector

`egg_size3`, a numeric vector

`status4`, a numeric vector

`dam4`, a numeric vector

`sire4`, a numeric vector

`tray4`, a numeric vector

`cell4`, a numeric vector

`egg_size4`, a numeric vector

`status5`, a numeric vector

`dam5`, a numeric vector

`sire5`, a numeric vector

`tray5`, a numeric vector

`cell5`, a numeric vector

`egg_size5`, a numeric vector

Source

Pitcher TE, Neff BD. 2007. Genetic quality and offspring performance in Chinook salmon: implications for supportive breeding. Conservation Genetics 8(3):607-616. DOI: 10.1007/s10592-006-9204-z

Examples

```
data(chinook_resampS)
survival_rcomp<- resampGlmer(resamp=chinook_resampS,dam="dam",sire="sire",response="status",
fam_link=binomial(logit),start=1,end=5) #full analysis should use 1,000 models
```

`chinook_survival` *Chinook salmon survival, raw data*

Description

Raw Chinook salmon numbers alive and dead to hatching of offspring produced using an 11 x 11 full factorial breeding design.

Usage

```
data("chinook_survival")
```

Format

A data frame with 242 observations on the following 9 variables.

`family`, a factor with levels: `f1 f10 f100 f101 f102 f103 f104 f105 f106 f107 f108 f109 f11 f110 f111 f112 f113 f114 f115 f116 f117 f118 f119 f12 f120 f121 f13 f14 f15 f16 f17 f18 f19 f2 f20 f21 f22 f23 f24 f25 f26 f27 f28 f29 f3 f30 f31 f32 f33 f34 f35 f36 f37 f38 f39 f4 f40 f41 f42 f43 f44 f45 f46 f47 f48 f49 f5 f50 f51 f52 f53 f54 f55 f56 f57 f58 f59 f6 f60 f61 f62 f63 f64 f65 f66 f67 f68 f69 f7 f70 f71 f72 f73 f74 f75 f76 f77 f78 f79 f8 f80 f81 f82 f83 f84 f85 f86 f87 f88 f89 f9 f90 f91 f92 f93 f94 f95 f96 f97 f98 f99`

`repli`, a factor with levels: `r1 r2`

`dam`, a factor with levels: `d1 d10 d11 d2 d3 d4 d5 d6 d7 d8 d9`

`sire`, a factor with levels: `s1 s10 s11 s2 s3 s4 s5 s6 s7 s8 s9`

`tray`, a factor with levels: `t1 t10 t11 t12 t13 t14 t15 t16 t2 t3 t4 t5 t6 t7 t8 t9`

`cell`, a factor with levels: `1A 1B 1C 1D 2A 2B 2C 2D 3A 3B 3C 3D 4A 4B 4C 4D`

`alive`, a numeric vector.

`dead`, a numeric vector.

`egg_size`, a numeric vector.

Details

Also includes family identity, family replicate, incubator position (tray and cell), and average female egg size (mm) information.

Source

http://link.springer.com.proxy1.lib.uwo.ca/article/10.1007

References

Pitcher TE, Neff BD. 2007. Genetic quality and offspring performance in Chinook salmon: implications for supportive breeding. Conservation Genetics 8(3):607-616. DOI: 10.1007/s10592-006-9204-z

Examples

```
data(chinook_survival)
## Convert replicate-level recorded data to individual-level (binary) data
chinook_survival2<- buildBinary(dat=chinook_survival,copy=c(2:6,9),one="alive",zero="dead")

## Standard additive genetic, non-additive genetic, and maternal variance analysis
survival_mod1<- observGlmer(observ=chinook_survival2,dam="dam",sire="sire",
response="status",fam_link=binomial(logit))
survival_mod1
```

`ciJack` *Jackknife confidence intervals*

Description

Extracts jackknife confidence intervals for additive genetic, non-additive genetic, and maternal variance components.

Usage

```
ciJack(comp, full, level = 95, rnd_r = 3, rnd_p = 1, trait = NULL)
```

Arguments

comp	Data frame of jackknife resampling results.
full	A vector of raw observed additive, non-additive, maternal, and total variance component values for from the full observed data set, i.e. c(additive, non-additive, maternal, total).
level	Confidence level, as a percentage. Default is 95.
rnd_r	Number of decimal places to round the confidence interval of raw values.
rnd_p	Number of decimal places to round the confidence interval of percentage values.
trait	Optional label for the phenotypic trait.

Details

Used for jackknife resampling results produced using *JackLmer* for normal data or *JackGlmer* for non-normal data. Jackknife confidence intervals, using pseudo-values are described by Efron and Tibshirani (1993). The standard errors are calculated from the pseudo-values and the Student's *t* distribution is used to provide the lower and upper confidence values. For delete-*d* jackknife resampling, *M* degrees of freedom are used for producing the confidence interval (Martin et al. 2004): $M = N / d$, where *N* is the total number of observations and d is the number of deleted observations. That is, *M* is the number of row in the jackknife resampling results. Large values of *M*, such as 1,000, can translate to the delete-d jackknife resampling method approaching bootstrap resampling expectations (Efron & Tibshirani 1993).

Value

Prints a data frame containing the lower, median, and upper values of the jackknife confidence interval for additive genetic, non-additive genetic, and maternal variance components. Values are presented as raw and percentages of the total variance value within each row.

References

Efron B, Tibshirani R. 1993. An introduction to the Bootstrap. Chapman and Hall, New York.

Martin, H., Westad, F. & Martens, H. (2004). Imporved Jackknife Variance Estimates of Bilinear Model Parameters. COMPSTAT 2004 – Proceedings in Computational Statistics 16th Symposium Held in Prague, Czech Republic, 2004 (ed J. Antoch), pp. 261-275. Physica-Verlag HD, Heidelberg.

See Also

ciJack2, ciJack3

Examples

```
data(chinook_jackL) #Chinook salmon offspring length, delete-one jackknife
ciJack(chinook_jackL,c(0.0000000,0.7192253,0.2029684,1.0404425))
```

`ciJack2` *Jackknife confidence intervals 2*

Description

Extracts jackknife confidence intervals for additive genetic, non-additive genetic, and maternal variance components. Also extracts intervals for optional position and block variance components.

Usage

```
ciJack2(comp, full, level = 95, rnd_r = 3, rnd_p = 1, position = NULL, block = NULL,
trait = NULL)
```

Arguments

`comp`	Data frame of jackknife resampling results.
`full`	A vector of raw observed additive, non-additive, maternal, and total variance component values for from the full observed data set, i.e. c(additive, non-additive, maternal, total, position/block). If there is a position and a block c(..., maternal, position, block).
`level`	Confidence level, as a percentage. Default is 95.
`rnd_r`	Number of decimal places to round the confidence interval of raw values.
`rnd_p`	Number of decimal places to round the confidence interval of percentage values.
`position`	Optional column name containing position factor information.
`block`	Optional column name containing block factor information.
`trait`	Optional label for the phenotypic trait.

Details

Used for jackknife resampling results produced using *JackLmer2* for normal data or *JackGlmer2* for non-normal data. Jackknife confidence intervals, using pseudo-values are described by Efron and Tibshirani (1993). The standard errors are calculated from the pseudo-values and the Student's t distribution is used to provide the lower and upper confidence values. For delete-d jackknife resampling, M degrees of freedom are used for producing the confidence interval (Martin et al. 2004): $M = N / d$, where N is the total number of observations and d is the number of deleted observations. That is, M is the number of row in the jackknife resampling results. Large values of M, such as 1,000, can translate to the delete-d jackknife resampling method approaching bootstrap resampling expectations (Efron & Tibshirani 1993).

Value

Prints a data frame containing the lower, median, and upper values of the jackknife confidence interval for additive genetic, non-additive genetic, maternal variance components, and optional position and/or block variance components. Values are presented as raw and percentages of the total variance value within each row.

References

Efron B, Tibshirani R. 1993. An introduction to the Bootstrap. Chapman and Hall, New York.

Martin, H., Westad, F. & Martens, H. (2004). Imporved Jackknife Variance Estimates of Bilinear Model Parameters. COMPSTAT 2004 – Proceedings in Computational Statistics 16th Symposium Held in Prague, Czech Republic, 2004 (ed J. Antoch), pp. 261-275. Physica-Verlag HD, Heidelberg.

See Also

ciJack, ciJack3

Examples

```
data(chinook_jackL) #Chinook salmon offspring length, delete-one jackknife
ciJack2(chinook_jackL,c(0.0000000,0.7192253,0.2029684,1.0404425,0.1077423),position="tray")
```

---

ciJack3 *Jackknife confidence intervals 3*

Description

Extracts jackknife confidence intervals for additive genetic, non-additive genetic, and maternal variance components. Also extracts intervals for additional fixed and random effects.

Usage

```
ciJack3(comp, full, remain = NULL, level = 95, rnd_r = 3, rnd_p = 1, trait = NULL)
```

Arguments

comp	Data frame of jackknife resampling results
full	A vector of raw observed additive, non-additive, maternal, and total variance component values for from the full observed data set, i.e. c(additive, non-additive, maternal, total). Followed by any other components in the order of the vector *remain*, i.e. c(additive, non-additive, maternal, total, component1, component2, ...).
remain	Vector of column names for additional effects
level	Confidence level, as a percentage. Default is 95.
rnd_r	Number of decimal places to round the confidence interval of raw values.
rnd_p	Number of decimal places to round the confidence interval of percentage values.
trait	Optional label for the phenotypic trait.

Details

Used for jackknife resampling results produced using *JackLmer3* for normal data or *JackGlmer3* for non-normal data. Jackknife confidence intervals, using pseudo-values are described by Efron and Tibshirani (1993). The standard errors are calculated from the pseudo-values and the Student's t distribution is used to provide the lower and upper confidence values. For delete-d jackknife resampling, M degrees of freedom are used for producing the confidence interval (Martin et al. 2004): $M = N / d$, where N is the total number of observations and d is the number of deleted observations. That is, M is the number of row in the jackknife resampling results. Large values of M, such as 1,000, can translate to the delete-d jackknife resampling method approaching bootstrap resampling expectations (Efron & Tibshirani 1993).

Value

Prints a data frame containing the lower, median, and upper values of the jackknife confidence interval for additive genetic, non-additive genetic, maternal variance components, and any additional fixed effect and random effect variance components. Values are presented as raw and percentages of the total variance value within each row.

References

Efron B, Tibshirani R. 1993. An introduction to the Bootstrap. Chapman and Hall, New York.

Martin, H., Westad, F. & Martens, H. (2004). Imporved Jackknife Variance Estimates of Bilinear Model Parameters. COMPSTAT 2004 – Proceedings in Computational Statistics 16th Symposium Held in Prague, Czech Republic, 2004 (ed J. Antoch), pp. 261-275. Physica-Verlag HD, Heidelberg.

See Also

ciJack, ciJack2

Examples

```
data(chinook_jackL) #Chinook salmon offspring length, delete-one jackknife
ciJack3(chinook_jackL,c(0.0000000,0.7192253,0.2029684,1.0404425,0.1077423,0.5499255),
remain=c("tray","Residual"))
```

---

ciMANA — *Bootstrap confidence intervals*

Description

Extracts bootstrap-*t* confidence intervals for additive genetic, non-additive genetic, and maternal variance components.

Usage

```
ciMANA(comp, level = 95, rnd_r = 3, rnd_p = 1, bias = NULL, accel = NULL, trait = NULL)
```

Arguments

comp	Data frame of bootstrap resampling results.
level	Confidence level, as a percentage. Default is 95.
rnd_r	Number of decimal places to round the confidence interval of raw values.
rnd_p	Number of decimal places to round the confidence interval of percentage values.
bias	Optional vector of raw observed additive, non-additive, and maternal, variance component values for bias correction, i.e. c(additive, non-additive, maternal, total).
accel	Optional data frame of jackknifed data model results for acceleration correction.
trait	Optional label for the phenotypic trait.

Details

Used for bootstrap resampling results produced using *resampLmer* for normal data or *resampGlmer* for non-normal data. Bootstrap-*t* confidence intervals, including bias and acceleration correction methods are described by Efron and Tibshirani (1993). Jackknife data model results for acceleration correction can be produced using *JackLmer*, for normal data or *JackGlmer* for non-normal data. The 'bias fail' warning is if the bias calculation is Inf or -Inf, e.g. *bias* contains a zero value, so the uncorrected confidence interval is displayed.

Value

Prints a data frame containing the lower, median, and upper values of the bootstrap-*t* confidence interval for additive genetic, non-additive genetic, and maternal variance components. Values are presented as raw and percentages of the total variance value within each row.

References

Efron B, Tibshirani R. 1993. An introduction to the Bootstrap. Chapman and Hall, New York.

See Also

ciMANA2, ciMANA3

Examples

```
#Import bootstrap resampled data model results
data(chinook_bootL) #Chinook salmon offspring length

#Extract un-corrected confidence interval
ciMANA(comp=chinook_bootL)

#Extract bias corrected confidence interval
ciMANA(comp=chinook_bootL,bias=c(0.0000000,0.7192253,0.2029684))
#see details for 'bias' fail

#Extract bias and accelerated corrected confidence interval
#Import jackknife resampled data model results
data(chinook_jackL)
#
ciMANA(comp=chinook_bootL,bias=c(0.0000000,0.7192253,0.2029684),accel=chinook_jackL)
#see details for 'bias' fail
```

---

ciMANA2 *Bootstrap confidence intervals 2*

Description

Extracts bootstrap-*t* confidence intervals for additive genetic, non-additive genetic, and maternal variance components. Also extracts intervals for optional position and block variance components.

Usage

```
ciMANA2(comp, level = 95, rnd_r = 3, rnd_p = 1, position = NULL, block = NULL,
bias = NULL, accel = NULL, trait = NULL)
```

Arguments

comp	Data frame of bootstrap resampling results.
level	Confidence level, as a percentage. Default is 95.
rnd_r	Number of decimal places to round the confidence interval of raw values.
rnd_p	Number of decimal places to round the confidence interval of percentage values.
position	Optional column name containing position factor information.
block	Optional column name containing block factor information.
bias	Optional vector of raw observed additive, non-additive, maternal, position and/or block variance component values for bias correction, i.e. c(additive, non-additive, maternal, position/block). If there is a position and a block c(..., maternal, position, block).
accel	Optional data frame of jackknifed data model results for acceleration correction.
trait	Optional label for the phenotypic trait.

Details

Used for bootstrap resampling results produced using *resampLmer2* for normal data or *resampGlmer2* for non-normal data. Bootstrap-*t* confidence intervals, including bias and acceleration correction methods are described by Efron and Tibshirani (1993). Jackknife data model results for acceleration correction can be produced using *JackLmer2*, for normal data or *JackGlmer2* for non-normal data. The 'bias fail' warning is if the bias calculation is Inf or -Inf, e.g. *bias* contains a zero value, so the uncorrected confidence interval is displayed.

Value

Prints a data frame containing the lower, median, and upper values of the bootstrap-*t* confidence interval for additive genetic, non-additive genetic, maternal, and optional position and/or block variance components. Values are presented as raw and percentages of the total variance value within each row.

References

Efron B, Tibshirani R. 1993. An introduction to the Bootstrap. Chapman and Hall, New York.

See Also

ciMANA, ciMANA3

Examples

```
#Import bootstrap resampled data model results
data(chinook_bootL) #Chinook salmon offspring length

#Extract un-corrected confidence interval
ciMANA2(comp=chinook_bootL,position="tray")
```

```
#Extract bias corrected confidence interval
ciMANA2(comp=chinook_bootL,bias=c(0.0000000,0.7192253,0.2029684,0.1077423),position="tray")
#see details for 'bias' fail

#Extract bias and accelerated corrected confidence interval
#Import jackknife resampled data model results
data(chinook_jackL)
#
ciMANA2(comp=chinook_bootL,bias=c(0.0000000,0.7192253,0.2029684,0.1077423),
accel=chinook_jackL,position="tray")
#see details for 'bias' fail
```

`ciMANA3` *Bootstrap confidence intervals 3*

Description

Extracts bootstrap-*t* confidence intervals for additive genetic, non-additive genetic, and maternal variance components. Also extracts intervals for additional fixed and random effects.

Usage

```
ciMANA3(comp, level = 95, rnd_r = 3, rnd_p = 1, bias = NULL, accel = NULL,
remain = NULL, trait = NULL)
```

Arguments

`comp`	Data frame of bootstrap resampling results.
`level`	Confidence level, as a percentage. Default is 95.
`rnd_r`	Number of decimal places to round the confidence interval of raw values.
`rnd_p`	Number of decimal places to round the confidence interval of percentage values.
`bias`	Optional vector of raw observed additive, non-additive, and maternal variance components for bias correction. Followed by any other components in the order of the vector *remain*, i.e. c(additive, non-additive, maternal, component1, component2, ...).
`accel`	Optional data frame of jackknifed data model results for acceleration correction.
`remain`	Vector of column names for additional effects.
`trait`	Optional label for the phenotypic trait.

Details

Used for bootstrap resampling results produced using *resampLmer3* for normal data or *resampGlmer3* for non-normal data. Bootstrap-*t* confidence intervals, including bias and acceleration correction methods are described by Efron and Tibshirani (1993). Jackknife data model results for acceleration correction can be produced using *JackLmer3*, for normal data or *JackGlmer3* for non-normal data. The 'bias fail' warning is if the bias calculation is Inf or -Inf, e.g. *bias* contains a zero value, so the uncorrected confidence interval is displayed.

Value

Prints a data frame containing the lower, median, and upper values of the bootstrap-*t* confidence interval for additive genetic, non-additive genetic, maternal, and any additional fixed effect and random effect variance components. Values are presented as raw and percentages of the total variance value within each row.

References

Efron B, Tibshirani R. 1993. An introduction to the Bootstrap. Chapman and Hall, New York.

See Also

ciMANA, ciMANA2

Examples

```
#Import bootstrap resampled data model results
data(chinook_bootL) #Chinook salmon offspring length

#Extract un-corrected confidence interval
ciMANA3(comp=chinook_bootL,remain=c("tray","Residual"))

#Extract bias corrected confidence interval
ciMANA3(comp=chinook_bootL,bias=c(0.0000000,0.7192253,0.2029684,0.1077423,0.5499255),
remain=c("tray","Residual"))
#see details for 'bias' fail

#Extract bias and accelerated corrected confidence interval
#Import jackknife resampled data model results
data(chinook_jackL)
#
ciMANA3(comp=chinook_bootL,bias=c(0.0000000,0.7192253,0.2029684,0.1077423,0.5499255),
accel=chinook_jackL,remain=c("tray","Residual"))
```

JackGlmer — *Jackknife components for non-normal data*

Description

Extracts additive genetic, non-additive genetic, and maternal variance components from a linear mixed-effect model using the *lmer* function of the *lme4* package. Model random effects are dam, sire, and dam by sire.

Usage

```
JackGlmer(observ, dam, sire, response, fam_link, quasi = F, size = 1, first = NULL)
```

Arguments

`observ`	Data frame of observed data.
`dam`	Column name containing dam(female) parent identity information.
`sire`	Column name containing sire(male) parent identity information.
`response`	Column name containing the offspring (response) phenotype values.
`fam_link`	The family and link in family(link) format. Supported options are binomial(logit), binomial(probit), poisson(log), and poisson (sqrt).
`quasi`	Incorporate overdispersion or quasi-error structure.
`size`	Default is 1 for delete-one jackknife resampling. If *size* > 1, delete-*d* jackknife resampling occurs removing a block d equal to *size*.
`first`	Number of initial sub-samples to run. Useful for examing if there is variation among sub-samples before jackknife resampling the entire data set. There can be little varitation for delete-one jackknife resampling with large data sets, and delete-*d* jackknife resampling should be considered.

Details

Uses delete-one jackknife resampling (Efron & Tibshirani 1993, p. 141-145). For the option of delete-*d* jackknife resampling, the rows of the observed data frame are shuffled and a block of observations of size *d* is deleted sequentially. Laplace approximation parameter estimation is used, which is a true likelihood method (Bolker et al. 2009). For the overdispersion option, an observation-level random effect is added to the model (Atkins et al. 2013). Extracts the dam, sire, dam, and dam by sire variance components. The residual variance component for the *fam_links* are described by Nakagawa and Schielzeth (2010, 2013). Calculates the total variance component. Calculates the additive genetic, non-additive genetic, and maternal variance components (see Lynch and Walsh 1998, p. 603).

Value

A data frame with columns containing the raw variance components for dam, sire, dam by sire, residual, total, additive genetic, non-additive genetic, and maternal. The number of rows in the data frame matches the total number of observations (*N*) for delete-one jackknife resampling or *M* groups for delete-*d* jackknife resampling to the lowest integer. Each row represents a deleted single observation or deleted *d* observations group.

Note

The Laplace approximation is used because there were fewer disadvantages relative to penalized quasi-likelihood and Gauss-Hermite quadrature parameter estimation (Bolker et al. 2009). That is, penalized quasi-likelihood is not recommended for count responses with means less than 5 and binary responses with less than 5 successes per group. Gauss-Hermite quadrature is not recommended for more than two or three random effects because of the rapidly declining analytical speed with the increasing number of random effects.

References

Atkins DC, Baldwin SA, Zheng C, Gallop RJ, Neighbors C. 2013. A tutorial on count regression and zero-altered count models for longitudinal substance use data. Psychology of Addictive Behaviors 27(1): 166-177. DOI: 10.1037/a0029508

Bolker BM, Brooks ME, Clark CJ, Geange SW, Poulsen JR, Stevens MHH, White J-SS. 2009. Generalized linear mixed models: a practical guide for ecology and evolution. Trends in Ecology and Evolution 24(3): 127-135. DOI: 10.1016/j.tree.2008.10.008

Efron B, Tibshirani R. 1993. An introduction to the Bootstrap. Chapman and Hall, New York.

Lynch M, Walsh B. 1998. Genetics and Analysis of Quantitative Traits. Sinauer Associates, Massachusetts.

Nakagawa S, Schielzeth H. 2010. Repeatability for Gaussian and non-Gaussian data: a practical guide for biologists. Biological Reviews 85(4): 935-956. DOI: 10.1111/j.1469-185X.2010.00141.x

Nakagawa S, Schielzeth H. 2013. A general and simple method for obtaining R2 from generalized linear mixed-effects models. Methods in Ecology and Evolution 4(2): 133-142. DOI: 10.1111/j.2041-210x.2012.00261.x

See Also

JackGlmer2, JackGlmer3

Examples

```
data(chinook_survival) #Chinook salmon offspring survival
## Convert replicate-level recorded data to individual-level (binary) data
chinook_survival2<- buildBinary(dat=chinook_survival,copy=c(1:6,9),one="alive",zero="dead")

#Delete-one
#survival_jack1<- JackGlmer(observ=chinook_survival2,dam="dam",sire="sire",
#response="status",fam_link=binomial(logit))
survival_jack1<- JackGlmer(observ=chinook_survival2,dam="dam",sire="sire",
response="status",fam_link=binomial(logit),first=2)  #first 2, a few minutes

#Delete-d, d=36
#survival_jack1.2<- JackGlmer(observ=chinook_survival2,dam="dam",sire="sire",
#response="status",fam_link=binomial(logit),size=36)
survival_jack1.2<- JackGlmer(observ=chinook_survival2,dam="dam",sire="sire",
response="status",fam_link=binomial(logit),size=36,first=2) #first 2, a few minutes
```

JackGlmer2 *Jackknife components for non-normal data 2*

Description

Extracts additive genetic, non-additive genetic, and maternal variance components from a linear mixed-effect model using the *lmer* function of the *lme4* package. Model random effects are dam, sire, and dam by sire. Options to include one random position and/or one random block effect(s).

Usage

```
JackGlmer2(observ, dam, sire, response, fam_link, position = NULL, block = NULL,
quasi = F, size = 1, first = NULL)
```

Arguments

`observ`	Data frame of observed data.
`dam`	Column name containing dam(female) parent identity information.
`sire`	Column name containing sire(male) parent identity information.
`response`	Column name containing the offspring (response) phenotype values.
`fam_link`	The family and link in family(link) format. Supported options are binomial(logit), binomial(probit), poisson(log), and poisson (sqrt).
`position`	Optional column name containing position factor information.
`block`	Optional column name containing block factor information.
`quasi`	Incorporate overdispersion or quasi-error structure.
`size`	Default is 1 for delete-one jackknife resampling. If *size* > 1, delete-*d* jackknife resampling occurs removing a block d equal to *size*.
`first`	Number of initial sub-samples to run. Useful for examing if there is variation among sub-samples before jackknife resampling the entire data set. There can be little varitation for delete-one jackknife resampling with large data sets, and delete-*d* jackknife resampling should be considered.

Details

Uses delete-one jackknife resampling (Efron & Tibshirani 1993, p. 141-145). For the option of delete-*d* jackknife resampling, the rows of the observed data frame are shuffled and a block of observations of size *d* is deleted sequentially. Laplace approximation parameter estimation is used, which is a true likelihood method (Bolker et al. 2009). For the overdispersion option, an observation-level random effect is added to the model (Atkins et al. 2013). Extracts the dam, sire, dam, and dam by sire variance components. Extracts optional position and block variance components. The residual variance component for the *fam_links* are described by Nakagawa and Schielzeth (2010, 2013). Calculates the total variance component. Calculates the additive genetic, non-additive genetic, and maternal variance components (see Lynch and Walsh 1998, p. 603).

Value

A data frame with columns containing the raw variance components for dam, sire, dam by sire, residual, total, additive genetic, non-additive genetic, and maternal. Also columns containing the raw variance components for the options of position and/or block. The number of rows in the data frame matches the total number of observations (*N*) for delete-one jackknife resampling or *M* groups for delete-*d* jackknife resampling to the lowest integer. Each row represents a deleted single observation or deleted *d* observations group.

Note

The Laplace approximation is used because there were fewer disadvantages relative to penalized quasi-likelihood and Gauss-Hermite quadrature parameter estimation (Bolker et al. 2009). That is, penalized quasi-likelihood is not recommended for count responses with means less than 5 and binary responses with less than 5 successes per group. Gauss-Hermite quadrature is not recommended for more than two or three random effects because of the rapidly declining analytical speed with the increasing number of random effects.

References

Atkins DC, Baldwin SA, Zheng C, Gallop RJ, Neighbors C. 2013. A tutorial on count regression and zero-altered count models for longitudinal substance use data. Psychology of Addictive Behaviors 27(1): 166-177. DOI: 10.1037/a0029508

Bolker BM, Brooks ME, Clark CJ, Geange SW, Poulsen JR, Stevens MHH, White J-SS. 2009. Generalized linear mixed models: a practical guide for ecology and evolution. Trends in Ecology and Evolution 24(3): 127-135. DOI: 10.1016/j.tree.2008.10.008

Efron B, Tibshirani R. 1993. An introduction to the Bootstrap. Chapman and Hall, New York.

Lynch M, Walsh B. 1998. Genetics and Analysis of Quantitative Traits. Sinauer Associates, Massachusetts.

Nakagawa S, Schielzeth H. 2010. Repeatability for Gaussian and non-Gaussian data: a practical guide for biologists. Biological Reviews 85(4): 935-956. DOI: 10.1111/j.1469-185X.2010.00141.x

Nakagawa S, Schielzeth H. 2013. A general and simple method for obtaining R2 from generalized linear mixed-effects models. Methods in Ecology and Evolution 4(2): 133-142. DOI: 10.1111/j.2041-210x.2012.00261.x

See Also

JackGlmer, JackGlmer3

Examples

```
data(chinook_survival) #Chinook salmon offspring survival
## Convert replicate-level recorded data to individual-level (binary) data
chinook_survival2<- buildBinary(dat=chinook_survival,copy=c(1:6,9),one="alive",zero="dead")

#Delete-one
#survival_jack2<- JackGlmer2(observ=chinook_survival2,dam="dam",sire="sire",
#response="status",fam_link=binomial(logit),position="tray")
survival_jack2<- JackGlmer2(observ=chinook_survival2,dam="dam",sire="sire",
response="status",fam_link=binomial(logit),position="tray",first=2)
#first 2, a few minutes

#Delete-d, d=36
#survival_jack2.2<- JackGlmer2(observ=chinook_survival2,dam="dam",sire="sire",
#response="status",fam_link=binomial(logit),position="tray",size=36)
survival_jack2.2<- JackGlmer2(observ=chinook_survival2,dam="dam",sire="sire",
response="status",fam_link=binomial(logit),position="tray",size=36,first=2)
#first 2, a few minutes
```

JackGlmer3 *Jackknife components for non-normal data 3*

Description

Extracts additive genetic, non-additive genetic, and maternal variance components from a linear mixed-effect model using the *lmer* function of the *lme4* package. Model random effects are dam, sire, dam by sire, and any additional fixed and/or random effects.

Usage

```
JackGlmer3(observ, dam, sire, response, fam_link, remain, quasi = F, size = 1,
first = NULL)
```

Arguments

observ	Data frame of observed data.
dam	Column name containing dam(female) parent identity information.
sire	Column name containing sire(male) parent identity information.
response	Column name containing the offspring (response) phenotype values.
fam_link	The family and link in family(link) format. Supported options are binomial(logit), binomial(probit), poisson(log), and poisson (sqrt).
remain	Remaining formula with # using *lme4* package formula.
quasi	Incorporate overdispersion or quasi-error structure.
size	Default is 1 for delete-one jackknife resampling. If *size* > 1, delete-*d* jackknife resampling occurs removing a block d equal to *size*.
first	Number of initial sub-samples to run. Useful for examing if there is variation among sub-samples before jackknife resampling the entire data set. There can be little varitation for delete-one jackknife resampling with large data sets, and delete-*d* jackknife resampling should be considered.

Details

Uses delete-one jackknife resampling (Efron & Tibshirani 1993, p. 141-145). For the option of delete-*d* jackknife resampling, the rows of the observed data frame are shuffled and a block of observations of size *d* is deleted sequentially. Laplace approximation parameter estimation is used, which is a true likelihood method (Bolker et al. 2009). For the overdispersion option, an observation-level random effect is added to the model (Atkins et al. 2013). Extracts the dam, sire, dam, and dam by sire variance components. Extracts any additional fixed effect and random effect variance components. The fixed-effect variance component is as a single group using the method described by Nakagawa and Schielzeth (2013). The residual variance component for the *fam_links* are described by Nakagawa and Schielzeth (2010, 2013). Calculates the total variance component. Calculates the additive genetic, non-additive genetic, and maternal variance components (see Lynch and Walsh 1998, p. 603).

Value

A data frame with columns containing the raw variance components for dam, sire, dam by sire, residual, total, additive genetic, non-additive genetic, and maternal. Also columns containing the raw variance components for remaining formula components. The number of rows in the data frame matches the total number of observations (*N*) for delete-one jackknife resampling or *M* groups for delete-*d* jackknife resampling to the lowest integer. Each row represents a deleted single observation or deleted *d* observations group.

Note

The Laplace approximation is used because there were fewer disadvantages relative to penalized quasi-likelihood and Gauss-Hermite quadrature parameter estimation (Bolker et al. 2009). That is, penalized quasi-likelihood is not recommended for count responses with means less than 5 and

binary responses with less than 5 successes per group. Gauss-Hermite quadrature is not recommended for more than two or three random effects because of the rapidly declining analytical speed with the increasing number of random effects.

References

Atkins DC, Baldwin SA, Zheng C, Gallop RJ, Neighbors C. 2013. A tutorial on count regression and zero-altered count models for longitudinal substance use data. Psychology of Addictive Behaviors 27(1): 166-177. DOI: 10.1037/a0029508

Bolker BM, Brooks ME, Clark CJ, Geange SW, Poulsen JR, Stevens MHH, White J-SS. 2009. Generalized linear mixed models: a practical guide for ecology and evolution. Trends in Ecology and Evolution 24(3): 127-135. DOI: 10.1016/j.tree.2008.10.008

Efron B, Tibshirani R. 1993. An introduction to the Bootstrap. Chapman and Hall, New York.

Lynch M, Walsh B. 1998. Genetics and Analysis of Quantitative Traits. Sinauer Associates, Massachusetts.

Nakagawa S, Schielzeth H. 2010. Repeatability for Gaussian and non-Gaussian data: a practical guide for biologists. Biological Reviews 85(4): 935-956. DOI: 10.1111/j.1469-185X.2010.00141.x

Nakagawa S, Schielzeth H. 2013. A general and simple method for obtaining R2 from generalized linear mixed-effects models. Methods in Ecology and Evolution 4(2): 133-142. DOI: 10.1111/j.2041-210x.2012.00261.x

See Also

JackGlmer, JackGlmer2

Examples

```
data(chinook_survival) #Chinook salmon offspring survival
## Convert replicate-level recorded data to individual-level (binary) data
chinook_survival2<- buildBinary(dat=chinook_survival,copy=c(1:6,9),one="alive",zero="dead")

#Delete-one
#survival_jack3<- JackGlmer3(observ=chinook_survival2,dam="dam",sire="sire",
#response="status",fam_link=binomial(logit),remain="egg_size + (1|tray)")
survival_jack3<- JackGlmer3(observ=chinook_survival2,dam="dam",sire="sire",
response="status",fam_link=binomial(logit),remain="egg_size + (1|tray)",first=2)
#first 2, a few minutes

#Delete-d, d=36
#survival_jack3.2<- JackGlmer3(observ=chinook_survival2,dam="dam",sire="sire",
#response="status",fam_link=binomial(logit),remain="egg_size + (1|tray)",size=36)
survival_jack3.2<- JackGlmer3(observ=chinook_survival2,dam="dam",sire="sire",
response="status",fam_link=binomial(logit),remain="egg_size + (1|tray)",size=36,first=2)
#first 2, a few minutes
```

JackLmer *Jackknife components for normal data*

Description

Extracts additive genetic, non-additive genetic, and maternal variance components from a linear mixed-effect model using the *lmer* function of the *lme4* package. Model random effects are dam, sire, and dam by sire.

Usage

```
JackLmer(observ, dam, sire, response, ml = F, size = 1, first = NULL)
```

Arguments

`observ`	Data frame of observed data.
`dam`	Column name containing dam(female) parent identity information.
`sire`	Column name containing sire(male) parent identity information.
`response`	Column name containing the offspring (response) phenotype values.
`ml`	Default is FALSE for restricted maximum likelihood. Change to TRUE for maximum likelihood.
`size`	Default is 1 for delete-one jackknife resampling. If *size* > 1, delete-*d* jackknife resampling occurs removing a block d equal to *size*.
`first`	Number of initial sub-samples to run. Useful for examing if there is variation among sub-samples before jackknife resampling the entire data set. There can be little varitation for delete-one jackknife resampling with large data sets, and delete-*d* jackknife resampling should be considered.

Details

Uses delete-one jackknife resampling (Efron & Tibshirani 1993, p. 141-145). For the option of delete-*d* jackknife resampling, the rows of the observed data frame are shuffled and a block of observations of size *d* is deleted sequentially. Extracts the dam, sire, dam, dam by sire, and residual variance components. Calculates the total variance component. Calculates the additive genetic, non-additive genetic, and maternal variance components (see Lynch and Walsh 1998, p. 603).

Value

A data frame with columns containing the raw variance components for dam, sire, dam by sire, residual, total, additive genetic, non-additive genetic, and maternal. The number of rows in the data frame matches the total number of observations (*N*) for delete-one jackknife resampling or *M* groups for delete-*d* jackknife resampling to the lowest integer. Each row represents a deleted single observation or deleted *d* observations group.

Note

Maximum likelihood (ML) estimates the parameters that maximize the likelihood of the observed data and has the advantage of using all the data and accounting for non-independence (Lynch and Walsh 1998, p. 779; Bolker et al. 2009). On the other hand, ML has the disadvantage of assuming that all fixed effects are known without error, producing a downward bias in the estimation of the residual variance component. This bias can be large if there are lots of fixed effects, especially if sample sizes are small. Restricted maximum likelihood (REML) has the advantage of not assuming the fixed effects are known and averages over the uncertainty, so there can be less bias in the estimation of the residual variance component. However, REML only maximizes a portion of the likelihood to estimate the effect parameters, but is the preferred method for analyzing large data sets with complex structure.

References

Efron B, Tibshirani R. 1993. An introduction to the Bootstrap. Chapman and Hall, New York.

Lynch M, Walsh B. 1998. Genetics and Analysis of Quantitative Traits. Sinauer Associates, Massachusetts.

See Also

JackLmer2, JackLmer3

Examples

```
data(chinook_length) #Chinook salmon offspring length

#Delete-one
#length_jack1<- JackLmer(observ=chinook_length,dam="dam",sire="sire",response="length")
length_jack1<- JackLmer(observ=chinook_length,dam="dam",sire="sire",response="length",
first=2) #first 2

#Delete-d, d=5
#length_jack1.2<- JackLmer(observ=chinook_length,dam="dam",sire="sire",response="length",
#size=5)
length_jack1.2<- JackLmer(observ=chinook_length,dam="dam",sire="sire",response="length",
size=5,first=2) #first 2
```

JackLmer2 — *Jackknife components for normal data 2*

Description

Extracts additive genetic, non-additive genetic, and maternal variance components from a linear mixed-effect model using the *lmer* function of the *lme4* package. Model random effects are dam, sire, and dam by sire. Options to include one random position and/or one random block effect(s).

Usage

```
JackLmer2(observ, dam, sire, response, position = NULL, block = NULL, ml = F, size = 1,
first = NULL)
```

Arguments

observ	Data frame of observed data.
dam	Column name containing dam(female) parent identity information.
sire	Column name containing sire(male) parent identity information.
response	Column name containing the offspring (response) phenotype values.
position	Optional column name containing position factor information.
block	Optional column name containing block factor information.
ml	Default is FALSE for restricted maximum likelihood. Change to TRUE for maximum likelihood.
size	Default is 1 for delete-one jackknife resampling. If *size* > 1, delete-*d* jackknife resampling occurs removing a block d equal to *size*.

`first` Number of initial sub-samples to run. Useful for examing if there is variation among sub-samples before jackknife resampling the entire data set. There can be little varitation for delete-one jackknife resampling with large data sets, and delete-*d* jackknife resampling should be considered.

Details

Uses delete-one jackknife resampling (Efron & Tibshirani 1993, p. 141-145). For the option of delete-*d* jackknife resampling, the rows of the observed data frame are shuffled and a block of observations of size *d* is deleted sequentially. Extracts the dam, sire, dam, dam by sire, and residual variance components. Extracts optional position and block variance components. Calculates the total variance component. Calculates the additive genetic, non-additive genetic, and maternal variance components (see Lynch and Walsh 1998, p. 603).

Value

A data frame with columns containing the raw variance components for dam, sire, dam by sire, residual, total, additive genetic, non-additive genetic, and maternal. Also columns containing the raw variance components for the options of position and/or block. The number of rows in the data frame matches the total number of observations (*N*) for delete-one jackknife resampling or *M* groups for delete-*d* jackknife resampling to the lowest integer. Each row represents a deleted single observation or deleted *d* observations group.

Note

Maximum likelihood (ML) estimates the parameters that maximize the likelihood of the observed data and has the advantage of using all the data and accounting for non-independence (Lynch and Walsh 1998, p. 779; Bolker et al. 2009). On the other hand, ML has the disadvantage of assuming that all fixed effects are known without error, producing a downward bias in the estimation of the residual variance component. This bias can be large if there are lots of fixed effects, especially if sample sizes are small. Restricted maximum likelihood (REML) has the advantage of not assuming the fixed effects are known and averages over the uncertainty, so there can be less bias in the estimation of the residual variance component. However, REML only maximizes a portion of the likelihood to estimate the effect parameters, but is the preferred method for analyzing large data sets with complex structure.

References

Efron B, Tibshirani R. 1993. An introduction to the Bootstrap. Chapman and Hall, New York.

Lynch M, Walsh B. 1998. Genetics and Analysis of Quantitative Traits. Sinauer Associates, Massachusetts.

See Also

JackLmer, JackLmer3

Examples

```
data(chinook_length) #Chinook salmon offspring length

#Delete-one
#length_jack2<- JackLmer2(observ=chinook_length,dam="dam",sire="sire",response="length",
#position="tray")
length_jack2<- JackLmer2(observ=chinook_length,dam="dam",sire="sire",response="length",
```

```
position="tray",first=2) #first 2

#Delete-d, d=5
#length_jack2.2<- JackLmer2(observ=chinook_length,dam="dam",sire="sire",response="length",
#position="tray",size=5)
length_jack2.2<- JackLmer2(observ=chinook_length,dam="dam",sire="sire",response="length",
position="tray",size=5,first=2) #first 2
```

JackLmer3 *Jackknife components for normal data 3*

Description

Extracts additive genetic, non-additive genetic, and maternal variance components from a linear mixed-effect model using the *lmer* function of the *lme4* package. Model random effects are dam, sire, dam by sire, and any additional fixed and/or random effects.

Usage

```
JackLmer3(observ, dam, sire, response, remain, ml = F, size = 1, first = NULL)
```

Arguments

observ	Data frame of observed data
dam	Column name containing dam(female) parent identity information.
sire	Column name containing sire(male) parent identity information.
response	Column name containing the offspring (response) phenotype values.
remain	Remaining formula with # using *lme4* package format.
ml	Default is FALSE for restricted maximum likelihood. Change to TRUE for maximum likelihood.
size	Default is 1 for delete-one jackknife resampling. If *size* > 1, delete-*d* jackknife resampling occurs removing a block d equal to *size*.
first	Number of initial sub-samples to run. Useful for examing if there is variation among sub-samples before jackknife resampling the entire data set. There can be little varitation for delete-one jackknife resampling with large data sets, and delete-*d* jackknife resampling should be considered.

Details

Uses delete-one jackknife resampling (Efron & Tibshirani 1993, p. 141-145). For the option of delete-*d* jackknife resampling, the rows of the observed data frame are shuffled and a block of observations of size *d* is deleted sequentially. Extracts the dam, sire, dam, dam by sire, and residual variance components. Extracts any additional fixed effect and random effect variance components. The fixed-effect variance component is as a single group using the method described by Nakagawa and Schielzeth (2013). Calculates the total variance component. Calculates the additive genetic, non-additive genetic, and maternal variance components (see Lynch and Walsh 1998, p. 603).

Value

A data frame with columns containing the raw variance components for dam, sire, dam by sire, residual, total, additive genetic, non-additive genetic, and maternal. Also columns containing the raw variance components for remaining formula components. The number of rows in the data frame matches the total number of observations (*N*) for delete-one jackknife resampling or *M* groups for delete-*d* jackknife resampling to the lowest integer. Each row represents a deleted single observation or deleted *d* observations group.

Note

Maximum likelihood (ML) estimates the parameters that maximize the likelihood of the observed data and has the advantage of using all the data and accounting for non-independence (Lynch and Walsh 1998, p. 779; Bolker et al. 2009). On the other hand, ML has the disadvantage of assuming that all fixed effects are known without error, producing a downward bias in the estimation of the residual variance component. This bias can be large if there are lots of fixed effects, especially if sample sizes are small. Restricted maximum likelihood (REML) has the advantage of not assuming the fixed effects are known and averages over the uncertainty, so there can be less bias in the estimation of the residual variance component. However, REML only maximizes a portion of the likelihood to estimate the effect parameters, but is the preferred method for analyzing large data sets with complex structure.

References

Efron B, Tibshirani R. 1993. An introduction to the Bootstrap. Chapman and Hall, New York.

Lynch M, Walsh B. 1998. Genetics and Analysis of Quantitative Traits. Sinauer Associates, Massachusetts.

Nakagawa S, Schielzeth H. 2013. A general and simple method for obtaining R2 from generalized linear mixed-effects models. Methods in Ecology and Evolution 4(2): 133-142. DOI: 10.1111/j.2041-210x.2012.00261.x

See Also

JackLmer, JackLmer2

Examples

```
data(chinook_length) #Chinook salmon offspring length

#Delete-one
#length_jack3<- JackLmer3(observ=chinook_length,dam="dam",sire="sire",response="length",
#remain="egg_size + (1|tray)")
length_jack3<- JackLmer3(observ=chinook_length,dam="dam",sire="sire",response="length",
remain="egg_size + (1|tray)",first=2) #first 2

#Delete-d, d=5
#length_jack3.2<- JackLmer3(observ=chinook_length,dam="dam",sire="sire",response="length",
#remain="egg_size + (1|tray)",size=5)
length_jack3.2<- JackLmer3(observ=chinook_length,dam="dam",sire="sire",response="length",
remain="egg_size + (1|tray)",size=5,first=2) #first 2
```

observGlmer — *Variance components for non-normal data*

Description

Extracts additive genetic, non-additive genetic, and maternal variance components from a generalized linear mixed-effect model using the *glmer* function of the *lme4* package. Model random effects are dam, sire, and dam by sire.

Usage

```
observGlmer(observ, dam, sire, response, fam_link, quasi = F)
```

Arguments

`observ`	Data frame of observed data.
`dam`	Column name containing dam(female) parent identity information.
`sire`	Column name containing sire(male) parent identity information.
`response`	Column name containing the offspring (response) phenotype values.
`fam_link`	The family and link in family(link) format. Supported options are binomial(logit), binomial(probit), poisson(log), and poisson (sqrt).
`quasi`	Incorporate overdispersion or quasi-error structure.

Details

Laplace approximation parameter estimation is used, which is a true likelihood method (Bolker et al. 2009). For the overdispersion option, an observation-level random effect is added to the model (Atkins et al. 2013). Extracts the dam, sire, dam, and dam by sire variance components. The residual variance component for the *fam_links* are described by Nakagawa and Schielzeth (2010, 2013). Calculates the total variance component. Calculates the additive genetic, non-additive genetic, and maternal variance components (see Lynch and Walsh 1998, p. 603). Significance values for the random effects are determined using likelihood ratio tests (Bolker et al. 2009).

Value

A list object containing the raw variance components, the variance components as a percentage of the total variance component. Also, contains the difference in AIC and BIC, and likelihood ratio test Chi-square and p-value for all random effects.

Note

The Laplace approximation is used because there were fewer disadvantages relative to penalized quasi-likelihood and Gauss-Hermite quadrature parameter estimation (Bolker et al. 2009). That is, penalized quasi-likelihood is not recommended for count responses with means less than 5 and binary responses with less than 5 successes per group. Gauss-Hermite quadrature is not recommended for more than two or three random effects because of the rapidly declining analytical speed with the increasing number of random effects.

References

Atkins DC, Baldwin SA, Zheng C, Gallop RJ, Neighbors C. 2013. A tutorial on count regression and zero-altered count models for longitudinal substance use data. Psychology of Addictive Behaviors 27(1): 166-177. DOI: 10.1037/a0029508

Bolker BM, Brooks ME, Clark CJ, Geange SW, Poulsen JR, Stevens MHH, White J-SS. 2009. Generalized linear mixed models: a practical guide for ecology and evolution. Trends in Ecology and Evolution 24(3): 127-135. DOI: 10.1016/j.tree.2008.10.008

Lynch M, Walsh B. 1998. Genetics and Analysis of Quantitative Traits. Sinauer Associates, Massachusetts.

Nakagawa S, Schielzeth H. 2010. Repeatability for Gaussian and non-Gaussian data: a practical guide for biologists. Biological Reviews 85(4): 935-956. DOI: 10.1111/j.1469-185X.2010.00141.x

Nakagawa S, Schielzeth H. 2013. A general and simple method for obtaining R2 from generalized linear mixed-effects models. Methods in Ecology and Evolution 4(2): 133-142. DOI: 10.1111/j.2041-210x.2012.00261.x

See Also

observGlmer2, observGlmer3

Examples

```
data(chinook_survival) #Chinook salmon offspring survival
## Convert replicate-level recorded data to individual-level (binary) data
chinook_survival2<- buildBinary(dat=chinook_survival,copy=c(2:6,9),one="alive",zero="dead")
#
survival_mod1<- observGlmer(observ=chinook_survival2,dam="dam",sire="sire",response="status",
fam_link=binomial(logit))  #a few minutes
survival_mod1
```

---

observGlmer2 — *Variance components for non-normal data 2*

Description

Extracts additive genetic, non-additive genetic, and maternal variance components from a generalized linear mixed-effect model using the *glmer* function of the *lme4* package. Model random effects are dam, sire, and dam by sire. Options to include one random position and/or one random block effect(s).

Usage

```
observGlmer2(observ, dam, sire, response, fam_link, position = NULL, block = NULL,
quasi = F)
```

Arguments

observ	Data frame of observed data.
dam	Column name containing dam(female) parent identity information.
sire	Column name containing sire(male) parent identity information.

response	Column name containing the offspring (response) phenotype values.
fam_link	The family and link in family(link) format. Supported options are binomial(logit), binomial(probit), poisson(log), and poisson (sqrt).
position	Optional column name containing position factor information.
block	Optional column name containing block factor information.
quasi	Incorporate overdispersion or quasi-error structure.

Details

Laplace approximation parameter estimation is used, which is a true likelihood method (Bolker et al. 2009). For the overdispersion option, an observation-level random effect is added to the model (Atkins et al. 2013). Extracts the dam, sire, dam, and dam by sire variance components. Extracts optional position and block variance components. The residual variance component for the *fam_links* are described by Nakagawa and Schielzeth (2010, 2013). Calculates the total variance component. Calculates the additive genetic, non-additive genetic, and maternal variance components (see Lynch and Walsh 1998, p. 603). Significance values for the random effects are determined using likelihood ratio tests (Bolker et al. 2009).

Value

A list object containing the raw variance components, the variance components as a percentage of the total variance component. Also, contains the difference in AIC and BIC, and likelihood ratio test Chi-square and p-value for all random effects.

Note

The Laplace approximation is used because there were fewer disadvantages relative to penalized quasi-likelihood and Gauss-Hermite quadrature parameter estimation (Bolker et al. 2009). That is, penalized quasi-likelihood is not recommended for count responses with means less than 5 and binary responses with less than 5 successes per group. Gauss-Hermite quadrature is not recommended for more than two or three random effects because of the rapidly declining analytical speed with the increasing number of random effects.

References

Atkins DC, Baldwin SA, Zheng C, Gallop RJ, Neighbors C. 2013. A tutorial on count regression and zero-altered count models for longitudinal substance use data. Psychology of Addictive Behaviors 27(1): 166-177. DOI: 10.1037/a0029508

Bolker BM, Brooks ME, Clark CJ, Geange SW, Poulsen JR, Stevens MHH, White J-SS. 2009. Generalized linear mixed models: a practical guide for ecology and evolution. Trends in Ecology and Evolution 24(3): 127-135. DOI: 10.1016/j.tree.2008.10.008

Lynch M, Walsh B. 1998. Genetics and Analysis of Quantitative Traits. Sinauer Associates, Massachusetts.

Nakagawa S, Schielzeth H. 2010. Repeatability for Gaussian and non-Gaussian data: a practical guide for biologists. Biological Reviews 85(4): 935-956. DOI: 10.1111/j.1469-185X.2010.00141.x

Nakagawa S, Schielzeth H. 2013. A general and simple method for obtaining R2 from generalized linear mixed-effects models. Methods in Ecology and Evolution 4(2): 133-142. DOI: 10.1111/j.2041-210x.2012.00261.x

See Also

observGlmer, observGlmer3

Examples

```
data(chinook_survival) #Chinook salmon offspring survival
## Convert replicate-level recorded data to individual-level (binary) data
chinook_survival2<- buildBinary(dat=chinook_survival,copy=c(2:6,9),one="alive",zero="dead")
#
survival_mod2<- observGlmer2(observ=chinook_survival2,dam="dam",sire="sire",
response="status",fam_link=binomial(logit),position="tray")  #a few minutes
survival_mod2
```

---

observGlmer3 *Variance components for non-normal data 3*

Description

Extracts additive genetic, non-additive genetic, and maternal variance components from a generalized linear mixed-effect model using the *glmer* function of the *lme4* package. Model random effects are dam, sire, dam by sire, and any additional fixed and/or random effects.

Usage

```
observGlmer3(observ, dam, sire, response, fam_link, remain, quasi = F, iter = 1000)
```

Arguments

observ	Data frame of observed data.
dam	Column name containing dam(female) parent identity information.
sire	Column name containing sire(male) parent identity information.
response	Column name containing the offspring (response) phenotype values.
fam_link	The family and link in family(link) format. Supported options are binomial(logit), binomial(probit), poisson(log), and poisson (sqrt).
remain	Remaining formula using *lme4* package format.
quasi	Incorporate overdispersion or quasi-error structure.
iter	Number of iterations for computing the parametric bootstrap significance value for any fixed effects.

Details

Laplace approximation parameter estimation is used, which is a true likelihood method (Bolker et al. 2009). For the overdispersion option, an observation-level random effect is added to the model (Atkins et al. 2013). Extracts the dam, sire, dam, and dam by sire variance components. Extracts any additional fixed effect and random effect variance components. The fixed-effect variance component is as a single group using the method described by Nakagawa and Schielzeth (2013). The residual variance component for the *fam_links* are described by Nakagawa and Schielzeth (2010, 2013). Calculates the total variance component. Calculates the additive genetic, non-additive genetic, and maternal variance components (see Lynch and Walsh 1998, p. 603). Significance values for the random effects are determined using likelihood ratio tests (Bolker et al. 2009). Significance values for any fixed effects are determined using likelihood ratio tests and a parametric bootstrap method (Bolker et al. 2009) from the *mixed* function of the *afex* package.

Value

A list object containing the raw variance components, the variance components as a percentage of the total variance component. Contains the difference in AIC and BIC, likelihood ratio test Chi-square and p-value for both random and fixed effects. Also contains the parametric bootstrap p-value for fixed effects.

Note

The Laplace approximation is used because there were fewer disadvantages relative to penalized quasi-likelihood and Gauss-Hermite quadrature parameter estimation (Bolker et al. 2009). That is, penalized quasi-likelihood is not recommended for count responses with means less than 5 and binary responses with less than 5 successes per group. Gauss-Hermite quadrature is not recommended for more than two or three random effects because of the rapidly declining analytical speed with the increasing number of random effects.

References

Atkins DC, Baldwin SA, Zheng C, Gallop RJ, Neighbors C. 2013. A tutorial on count regression and zero-altered count models for longitudinal substance use data. Psychology of Addictive Behaviors 27(1): 166-177. DOI: 10.1037/a0029508

Bolker BM, Brooks ME, Clark CJ, Geange SW, Poulsen JR, Stevens MHH, White J-SS. 2009. Generalized linear mixed models: a practical guide for ecology and evolution. Trends in Ecology and Evolution 24(3): 127-135. DOI: 10.1016/j.tree.2008.10.008

Lynch M, Walsh B. 1998. Genetics and Analysis of Quantitative Traits. Sinauer Associates, Massachusetts.

Nakagawa S, Schielzeth H. 2010. Repeatability for Gaussian and non-Gaussian data: a practical guide for biologists. Biological Reviews 85(4): 935-956. DOI: 10.1111/j.1469-185X.2010.00141.x

Nakagawa S, Schielzeth H. 2013. A general and simple method for obtaining R2 from generalized linear mixed-effects models. Methods in Ecology and Evolution 4(2): 133-142. DOI: 10.1111/j.2041-210x.2012.00261.x

See Also

observGlmer, observGlmer2

Examples

```
data(chinook_survival) #Chinook salmon offspring survival
## Convert replicate-level recorded data to individual-level (binary) data
chinook_survival2<- buildBinary(dat=chinook_survival,copy=c(2:6,9),one="alive",zero="dead")
#just a few iterations for the p-value of fixed effect
survival_mod3<- observGlmer3(observ=chinook_survival2,dam="dam",sire="sire",
response="status",fam_link=binomial(logit),remain="egg_size + (1|tray)",iter=5)
#a few minutes

survival_mod3
```

`observLmer` *Variance components for normal data*

Description

Extracts additive genetic, non-additive genetic, and maternal variance components from a linear mixed-effect model using the *lmer* function of the *lme4* package. Model random effects are dam, sire, and dam by sire.

Usage

```
observLmer(observ, dam, sire, response, ml = F)
```

Arguments

`observ`	Data frame of observed data.
`dam`	Column name containing dam(female) parent identity information.
`sire`	Column name containing sire(male) parent identity information.
`response`	Column name containing the offspring (response) phenotype values.
`ml`	Default is FALSE for restricted maximum likelihood. Change to TRUE for maximum likelihood.

Details

Extracts the dam, sire, dam, dam by sire, and residual variance components. Calculates the total variance component. Calculates the additive genetic, non-additive genetic, and maternal variance components (see Lynch and Walsh 1998, p. 603). Significance values for the random effects are determined using likelihood ratio tests (Bolker et al. 2009).

Value

A list object containing the raw variance components, the variance components as a percentage of the total variance component. Also, contains the difference in AIC and BIC, and likelihood ratio test Chi-square and p-value for all random effects.

Note

Maximum likelihood (ML) estimates the parameters that maximize the likelihood of the observed data and has the advantage of using all the data and accounting for non-independence (Lynch and Walsh 1998, p. 779; Bolker et al. 2009). On the other hand, ML has the disadvantage of assuming that all fixed effects are known without error, producing a downward bias in the estimation of the residual variance component. This bias can be large if there are lots of fixed effects, especially if sample sizes are small. Restricted maximum likelihood (REML) has the advantage of not assuming the fixed effects are known and averages over the uncertainty, so there can be less bias in the estimation of the residual variance component. However, REML only maximizes a portion of the likelihood to estimate the effect parameters, but is the preferred method for analyzing large data sets with complex structure.

References

Bolker BM, Brooks ME, Clark CJ, Geange SW, Poulsen JR, Stevens MHH, White J-SS. 2009. Generalized linear mixed models: a practical guide for ecology and evolution. Trends in Ecology and Evolution 24(3): 127-135. DOI: 10.1016/j.tree.2008.10.008

Lynch M, Walsh B. 1998. Genetics and Analysis of Quantitative Traits. Sinauer Associates, Massachusetts.

See Also

observLmer2, observLmer3

Examples

```
data(chinook_length) #Chinook salmon offspring length
length_mod1<- observLmer(observ=chinook_length,dam="dam",sire="sire",response="length")
length_mod1
```

---

observLmer2 *Variance components for normal data 2*

Description

Extracts additive genetic, non-additive genetic, and maternal variance components from a linear mixed-effect model using the *lmer* function of the *lme4* package. Model random effects are dam, sire, and dam by sire. Options to include one random position and/or one random block effect(s).

Usage

```
observLmer2(observ, dam, sire, response, position = NULL, block = NULL, ml = F)
```

Arguments

observ	Data frame of observed data.
dam	Column name containing dam(female) parent identity information.
sire	Column name containing sire(male) parent identity information.
response	Column name containing the offspring (response) phenotype values.
position	Optional column name containing position factor information.
block	Optional column name containing block factor information.
ml	Default is FALSE for restricted maximum likelihood. Change to TRUE for maximum likelihood.

Details

Extracts the dam, sire, dam, dam by sire, and residual variance components. Extracts optional position and block variance components. Calculates the total variance component. Calculates the additive genetic, non-additive genetic, and maternal variance components (see Lynch and Walsh 1998, p. 603). Significance values for the random effects are determined using likelihood ratio tests (Bolker et al. 2009).

Value

A list object containing the raw variance components, the variance components as a percentage of the total variance component. Also, contains the difference in AIC and BIC, and likelihood ratio test Chi-square and p-value for all random effects.

Note

Maximum likelihood (ML) estimates the parameters that maximize the likelihood of the observed data and has the advantage of using all the data and accounting for non-independence (Lynch and Walsh 1998, p. 779; Bolker et al. 2009). On the other hand, ML has the disadvantage of assuming that all fixed effects are known without error, producing a downward bias in the estimation of the residual variance component. This bias can be large if there are lots of fixed effects, especially if sample sizes are small. Restricted maximum likelihood (REML) has the advantage of not assuming the fixed effects are known and averages over the uncertainty, so there can be less bias in the estimation of the residual variance component. However, REML only maximizes a portion of the likelihood to estimate the effect parameters, but is the preferred method for analyzing large data sets with complex structure.

References

Bolker BM, Brooks ME, Clark CJ, Geange SW, Poulsen JR, Stevens MHH, White J-SS. 2009. Generalized linear mixed models: a practical guide for ecology and evolution. Trends in Ecology and Evolution 24(3): 127-135. DOI: 10.1016/j.tree.2008.10.008

Lynch M, Walsh B. 1998. Genetics and Analysis of Quantitative Traits. Sinauer Associates, Massachusetts.

See Also

observLmer, observLmer3

Examples

```
data(chinook_length) #Chinook salmon offspring length
length_mod2<- observLmer2(observ=chinook_length,dam="dam",sire="sire",response="length",
position="tray")
length_mod2
```

---

observLmer3 *Variance components for normal data 3*

Description

Extracts additive genetic, non-additive genetic, and maternal variance components from a linear mixed-effect model using the *lmer* function of the *lme4* package. Model random effects are dam, sire, dam by sire, and any additional fixed and/or random effects.

Usage

```
observLmer3(observ, dam, sire, response, remain, ml = F, iter = 1000)
```

Arguments

`observ`	Data frame of observed data.
`dam`	Column name containing dam(female) parent identity information.
`sire`	Column name containing sire(male) parent identity information.
`response`	Column name containing the offspring (response) phenotype values.
`remain`	Remaining formula using *lme4* package format.
`ml`	Default is FALSE for restricted maximum likelihood. Change to TRUE for maximum likelihood.
`iter`	Number of iterations for computing the parametric bootstrap significance value for any fixed effects.

Details

Extracts the dam, sire, dam, dam by sire, and residual variance components. Extracts any additional fixed effect and random effect variance components. The fixed-effect variance component is as a single group using the method described by Nakagawa and Schielzeth (2013). Calculates the total variance component. Calculates the additive genetic, non-additive genetic, and maternal variance components (see Lynch and Walsh 1998, p. 603). Significance values for the random effects are determined using likelihood ratio tests (Bolker et al. 2009). Significance values for any fixed effects are determined using likelihood ratio tests and a parametric bootstrap method (Bolker et al. 2009) from the *mixed* function of the *afex* package.

Value

A list object containing the raw variance components, the variance components as a percentage of the total variance component. Contains the difference in AIC and BIC, likelihood ratio test Chi-square and p-value for both random and fixed effects. Also contains the parametric bootstrap p-value for fixed effects.

Note

Maximum likelihood (ML) estimates the parameters that maximize the likelihood of the observed data and has the advantage of using all the data and accounting for non-independence (Lynch and Walsh 1998, p. 779; Bolker et al. 2009). On the other hand, ML has the disadvantage of assuming that all fixed effects are known without error, producing a downward bias in the estimation of the residual variance component. This bias can be large if there are lots of fixed effects, especially if sample sizes are small. Restricted maximum likelihood (REML) has the advantage of not assuming the fixed effects are known and averages over the uncertainty, so there can be less bias in the estimation of the residual variance component. However, REML only maximizes a portion of the likelihood to estimate the effect parameters, but is the preferred method for analyzing large data sets with complex structure.

References

Bolker BM, Brooks ME, Clark CJ, Geange SW, Poulsen JR, Stevens MHH, White J-SS. 2009. Generalized linear mixed models: a practical guide for ecology and evolution. Trends in Ecology and Evolution 24(3): 127-135. DOI: 10.1016/j.tree.2008.10.008

Lynch M, Walsh B. 1998. Genetics and Analysis of Quantitative Traits. Sinauer Associates, Massachusetts.

Nakagawa S, Schielzeth H. 2013. A general and simple method for obtaining R2 from generalized linear mixed-effects models. Methods in Ecology and Evolution 4(2): 133-142. DOI: 10.1111/j.2041-210x.2012.00261.x

See Also

observLmer, observLmer2

Examples

```
data(chinook_length) #Chinook salmon offspring length
#just a few iterations for the p-value of fixed effect
length_mod3<- observLmer3(observ=chinook_length,dam="dam",sire="sire",response="length",
remain="egg_size + (1|tray)",iter=5)
length_mod3
```

---

powerGlmer *Power analysis for non-normal data*

Description

Extracts the power values of dam, sire, and dam by sire variance components from a generalized linear mixed-effect model using the *glmer* function of the *lme4* package.

Usage

```
powerGlmer(varcomp, nval, fam_link, alpha = 0.05, nsim = 100, poisLog = NULL)
```

Arguments

varcomp	Vector of known dam, sire, and dam by sire variance components, i.e. c(dam,sire,ds).
nval	Vector of known dam, sire, and offspring per family sample sizes, i.e. c(dam,sire, offspring).
fam_link	The family and link in family(link) format. Supported options are binomial(logit), binomial(probit), poisson(log), and poisson (sqrt).
alpha	Statistical significance value. Default is 0.05.
nsim	Number of simulations. Default is 100.
poisLog	Known poisson(log) variance component value.

Details

Extracts the dam, sire, dam, and dam by sire power values. The residual variance component for the *fam_links* are described by Nakagawa and Schielzeth (2010, 2013). Power values are calculated by stochastically simulation data and then calculating the proportion of significance values less than *alpha* for each component (Bolker 2008). Significance values for the random effects are determined using likelihood ratio tests (Bolker et al. 2009).

Value

A data frame with the sample sizes, variance component inputs, variance component outputs, and power values.

Note

The Laplace approximation is used because there were fewer disadvantages relative to penalized quasi-likelihood and Gauss-Hermite quadrature parameter estimation (Bolker et al. 2009). That is, penalized quasi-likelihood is not recommended for count responses with means less than 5 and binary responses with less than 5 successes per group. Gauss-Hermite quadrature is not recommended for more than two or three random effects because of the rapidly declining analytical speed with the increasing number of random effects.

References

Bolker BM. 2008. Ecological models and data in R. Princeton University Press, New Jersey.

Bolker BM, Brooks ME, Clark CJ, Geange SW, Poulsen JR, Stevens MHH, White J-SS. 2009. Generalized linear mixed models: a practical guide for ecology and evolution. Trends in Ecology and Evolution 24(3): 127-135. DOI: 10.1016/j.tree.2008.10.008

Lynch M, Walsh B. 1998. Genetics and Analysis of Quantitative Traits. Sinauer Associates, Massachusetts.

Nakagawa S, Schielzeth H. 2010. Repeatability for Gaussian and non-Gaussian data: a practical guide for biologists. Biological Reviews 85(4): 935-956. DOI: 10.1111/j.1469-185X.2010.00141.x

Nakagawa S, Schielzeth H. 2013. A general and simple method for obtaining R2 from generalized linear mixed-effects models. Methods in Ecology and Evolution 4(2): 133-142. DOI: 10.1111/j.2041-210x.2012.00261.x

See Also

powerGlmer2, powerGlmer3

Examples

```
#100 simulations
#pwr_G1<- powerGlmer(varcomp=c(1,0.15,0.11),nval=c(10,10,20),fam_link=binomial(logit))
#pwr_G1
#5 simulations
pwr_G1<- powerGlmer(varcomp=c(1,0.15,0.11),nval=c(10,10,20),fam_link=binomial(logit),
nsim=5)
pwr_G1
```

---

powerGlmer2 *Power analysis for non-normal data 2*

Description

Extracts the power values of dam, sire, and dam by sire variance components from a generalized linear mixed-effect model using the *glmer* function of the *lme4* package. Options to include one random position and/or one random block effect(s).

Usage

```
powerGlmer2(varcomp, nval, fam_link, alpha = 0.05, nsim = 100, position = NULL,
block = NULL, poisLog = NULL)
```

Arguments

`varcomp`	Vector of known dam, sire, dam by sire, and position and/or block variance components, i.e. c(dam,sire,ds,position/block). If there is a position and a block c(..., ds, position, block).
`nval`	Vector of known dam, sire, offspring per family, and position and/or block sample sizes, i.e. c(dam,sire,offspring,position/block). If there is a position and a block c(..., offspring, position, block).
`fam_link`	The family and link in family(link) format. Supported options are binomial(logit), binomial(probit), poisson(log), and poisson (sqrt).
`alpha`	Statistical significance value. Default is 0.05.
`nsim`	Number of simulations. Default is 100.
`position`	Optional number of replicates to divide the number of offspring for the number of positions.
`block`	Optional vector of dams and sires per block, e.g. c(2,2).
`poisLog`	Known poisson(log) variance component value.

Details

Extracts the dam, sire, dam, dam by sire, and position and/or block power values. The residual variance component for the *fam_links* are described by Nakagawa and Schielzeth (2010, 2013). Power values are calculated by stochastically simulation data and then calculating the proportion of significance values less than *alpha* for each component (Bolker 2008). Significance values for the random effects are determined using likelihood ratio tests (Bolker et al. 2009).

Value

A data frame with the sample sizes, variance component inputs, variance component outputs, and power values.

Note

The Laplace approximation is used because there were fewer disadvantages relative to penalized quasi-likelihood and Gauss-Hermite quadrature parameter estimation (Bolker et al. 2009). That is, penalized quasi-likelihood is not recommended for count responses with means less than 5 and binary responses with less than 5 successes per group. Gauss-Hermite quadrature is not recommended for more than two or three random effects because of the rapidly declining analytical speed with the increasing number of random effects.

References

Bolker BM. 2008. Ecological models and data in R. Princeton University Press, New Jersey.

Bolker BM, Brooks ME, Clark CJ, Geange SW, Poulsen JR, Stevens MHH, White J-SS. 2009. Generalized linear mixed models: a practical guide for ecology and evolution. Trends in Ecology and Evolution 24(3): 127-135. DOI: 10.1016/j.tree.2008.10.008

Lynch M, Walsh B. 1998. Genetics and Analysis of Quantitative Traits. Sinauer Associates, Massachusetts.

Nakagawa S, Schielzeth H. 2010. Repeatability for Gaussian and non-Gaussian data: a practical guide for biologists. Biological Reviews 85(4): 935-956. DOI: 10.1111/j.1469-185X.2010.00141.x

Nakagawa S, Schielzeth H. 2013. A general and simple method for obtaining R2 from generalized linear mixed-effects models. Methods in Ecology and Evolution 4(2): 133-142. DOI: 10.1111/j.2041-210x.2012.00261.x

See Also

powerGlmer, powerGlmer3

Examples

```
#100 simulations
#pwr_G2<- powerGlmer2(varcomp=c(1,0.15,0.11,0.5),nval=c(10,10,20,10),
#fam_link=binomial(logit),position=2)
#pwr_G2
#5 simulations
pwr_G2<- powerGlmer2(varcomp=c(1,0.15,0.11,0.5),nval=c(10,10,20,10),
fam_link=binomial(logit),position=2,nsim=5)
pwr_G2
```

---

powerGlmer3 *Power analysis for non-normal data 3*

Description

Extracts the power values of dam, sire, and dam by sire variance components from a generalized linear mixed-effect model using the *glmer* function of the *lme4* package. Model can include additional fixed and/or random effects.

Usage

```
powerGlmer3(var_rand, n_rand, design, remain, fam_link, var_fix = NULL, n_fix = NULL,
 alpha = 0.05, nsim = 100, poisLog = NULL, ftest = "LR", iter = NULL)
```

Arguments

var_rand	Vector of known dam, sire, dam by sire, and remaining random variance components, i.e. c(dam,sire,ds,rand1,rand2,...).
n_rand	Vector of known dam, sire, family, and remaining random sample sizes, i.e. c(dam,sire,family,rand1,rand2,...).
design	A data frame of the experimental design, using only integers. First three columns must contain and be named "dam", "sire", "family". Remaining columns are the random effects followed by the fixed effects. Continuous fixed effects are a column containing the values 1:nrow(design).
remain	Remaining formula using *lme4* package format. Must be random effects followed by fixed effects. No interactions or random slopes; formulate as intercepts in design.
fam_link	The family and link in family(link) format. Supported options are binomial(logit), binomial(probit), poisson(log), and poisson (sqrt).
var_fix	Vector of known fixed variance components, i.e. c(fix1,fix2,...). Continous fixed random values are sorted to match column values.
n_fix	Vector of known fixed sample sizes, i.e. c(fix1,fix2,...). Continuous fixed effects must have a sample size of 1.
alpha	Statistical significance value. Default is 0.05.
nsim	Number of simulations. Default is 100.

`poisLog` Known poisson(log) variance component value.

`ftest` Default is "LR" for likelihood ratio test for fixed effects. Option "PB" is for parametric bootstrap.

`iter` Number of iterations for computing the parametric bootstrap significance value for any fixed effects.

Details

Extracts the dam, sire, dam, dam by sire, and any remaining random and fixed effects power values. The residual variance component for the *fam_links* are described by Nakagawa and Schielzeth (2010, 2013). Power values are calculated by stochastically simulation data and then calculating the proportion of significance values less than *alpha* for each component (Bolker 2008). Significance values for the random effects are determined using likelihood ratio tests (Bolker et al. 2009). Significance values for any fixed effects are determined using likelihood ratio tests or parametric bootstrap method (Bolker et al. 2009) from the *mixed* function of the *afex* package.

Value

A data frame with the sample sizes, variance component inputs, variance component outputs, and power values.

Note

The Laplace approximation is used because there were fewer disadvantages relative to penalized quasi-likelihood and Gauss-Hermite quadrature parameter estimation (Bolker et al. 2009). That is, penalized quasi-likelihood is not recommended for count responses with means less than 5 and binary responses with less than 5 successes per group. Gauss-Hermite quadrature is not recommended for more than two or three random effects because of the rapidly declining analytical speed with the increasing number of random effects.

References

Bolker BM. 2008. Ecological models and data in R. Princeton University Press, New Jersey.

Bolker BM, Brooks ME, Clark CJ, Geange SW, Poulsen JR, Stevens MHH, White J-SS. 2009. Generalized linear mixed models: a practical guide for ecology and evolution. Trends in Ecology and Evolution 24(3): 127-135. DOI: 10.1016/j.tree.2008.10.008

Lynch M, Walsh B. 1998. Genetics and Analysis of Quantitative Traits. Sinauer Associates, Massachusetts.

Nakagawa S, Schielzeth H. 2010. Repeatability for Gaussian and non-Gaussian data: a practical guide for biologists. Biological Reviews 85(4): 935-956. DOI: 10.1111/j.1469-185X.2010.00141.x

Nakagawa S, Schielzeth H. 2013. A general and simple method for obtaining R2 from generalized linear mixed-effects models. Methods in Ecology and Evolution 4(2): 133-142. DOI: 10.1111/j.2041-210x.2012.00261.x

See Also

`powerGlmer`, `powerGlmer2`

Examples

```
##design object: 2 remaining random effects and 1 continous fixed effect
block=c(2,2); blocN=10; position=2; posN=10; offN=20
dam0<- stack(as.data.frame(matrix(1:(block[1]*blocN),ncol=blocN,nrow=block[1])))
sire0<- stack(as.data.frame(matrix(1:(block[2]*blocN),ncol=blocN,nrow=block[2])))
observ0<- merge(dam0,sire0, by="ind")
levels(observ0[,1])<- 1:blocN; colnames(observ0)<- c("block","dam","sire")
observ0$family<- 1:nrow(observ0)  #add family
#expand for position
observ1<- do.call("rbind", replicate(position,observ0,simplify=FALSE));rm(observ0)
observ1$position<- sample(rep(1:posN,each=position)) #random assignment
#expand for offspring
observ<- do.call("rbind", replicate(offN,observ1,simplify=FALSE)); rm(observ1)
desn<- observ[,c(2,3,4,5,1)];rm(observ) #dam,sire,family,position,block
desn$egg_size<- 1:nrow(desn)
colnames(desn)[6]<- "egg_size"

#100 simulations
#pwr_G3<- powerGlmer3(var_rand=c(1,0.15,0.11,0.5,0.3),n_rand=c(20,20,40,10,10),
#fam_link=binomial(logit),var_fix=0.1,n_fix=1,design=desn,
#remain="(1|position)+(1|block)+egg_size")
#pwr_G3
#5 simulations
pwr_G3<- powerGlmer3(var_rand=c(1,0.15,0.11,0.5,0.3),n_rand=c(20,20,40,10,10),
fam_link=binomial(logit),var_fix=0.1,n_fix=1,design=desn,
remain="(1|position)+(1|block)+egg_size",nsim=5)
pwr_G3
```

---

powerLmer *Power analysis for normal data*

Description

Extracts the power values of dam, sire, and dam by sire variance components from a linear mixed-effect model using the *lmer* function of the *lme4* package.

Usage

```
powerLmer(varcomp, nval, alpha = 0.05, nsim = 100, ml = F)
```

Arguments

varcomp	Vector of known dam, sire, dam by sire, and residual variance components, i.e. c(dam,sire,ds,res).
nval	Vector of known dam, sire, and offspring per family sample sizes, i.e. c(dam,sire, offspring).
alpha	Statistical significance value. Default is 0.05.
nsim	Number of simulations. Default is 100.
ml	Default is FALSE for restricted maximum likelihood. Change to TRUE for maximum likelihood.

Details

Extracts the dam, sire, dam, and dam by sire power values. Power values are calculated by stochastically simulation data and then calculating the proportion of significance values less than *alpha* for each component (Bolker 2008). Significance values for the random effects are determined using likelihood ratio tests (Bolker et al. 2009).

Value

A data frame with the sample sizes, variance component inputs, variance component outputs, and power values.

Note

Maximum likelihood (ML) estimates the parameters that maximize the likelihood of the observed data and has the advantage of using all the data and accounting for non-independence (Lynch and Walsh 1998, p. 779; Bolker et al. 2009). On the other hand, ML has the disadvantage of assuming that all fixed effects are known without error, producing a downward bias in the estimation of the residual variance component. This bias can be large if there are lots of fixed effects, especially if sample sizes are small. Restricted maximum likelihood (REML) has the advantage of not assuming the fixed effects are known and averages over the uncertainty, so there can be less bias in the estimation of the residual variance component. However, REML only maximizes a portion of the likelihood to estimate the effect parameters, but is the preferred method for analyzing large data sets with complex structure.

References

Bolker BM. 2008. Ecological models and data in R. Princeton University Press, New Jersey.

Bolker BM, Brooks ME, Clark CJ, Geange SW, Poulsen JR, Stevens MHH, White J-SS. 2009. Generalized linear mixed models: a practical guide for ecology and evolution. Trends in Ecology and Evolution 24(3): 127-135. DOI: 10.1016/j.tree.2008.10.008

Lynch M, Walsh B. 1998. Genetics and Analysis of Quantitative Traits. Sinauer Associates, Massachusetts.

See Also

powerLmer2, powerLmer3

Examples

```
#100 simulations
#pwr_L1<- powerLmer(varcomp=c(0.19,0.03,0.02,0.76),nval=c(10,10,20))
#pwr_L1
#5simulations
pwr_L1<- powerLmer(varcomp=c(0.19,0.03,0.02,0.76),nval=c(10,10,20),nsim=5)
pwr_L1
```

powerLmer2	*Power analysis for normal data 2*

Description

Extracts the power values of dam, sire, and dam by sire variance components from a linear mixed-effect model using the *lmer* function of the *lme4* package. Options to include one random position and/or one random block effect(s).

Usage

```
powerLmer2(varcomp, nval, alpha = 0.05, nsim = 100, position = NULL, block = NULL,
ml = F)
```

Arguments

`varcomp`	Vector of known dam, sire, dam by sire, residual, and position and/or block variance components, i.e. c(dam,sire,ds,res,position/block). If there is a position and a block c(..., res, position, block).
`nval`	Vector of known dam, sire, offspring per family, and position and/or block sample sizes, i.e. c(dam,sire,offspring,position/block). If there is a position and a block c(..., offspring, position, block).
`alpha`	Statistical significance value. Default is 0.05.
`nsim`	Number of simulations. Default is 100.
`position`	Optional number of replicates to divide the number of offspring for the number of positions.
`block`	Optional vector of dams and sires per block, e.g. c(2,2).
`ml`	Default is FALSE for restricted maximum likelihood. Change to TRUE for maximum likelihood.

Details

Extracts the dam, sire, dam, dam by sire, and position and/or block power values. Power values are calculated by stochastically simulation data and then calculating the proportion of significance values less than *alpha* for each component (Bolker 2008). Significance values for the random effects are determined using likelihood ratio tests (Bolker et al. 2009).

Value

A data frame with the sample sizes, variance component inputs, variance component outputs, and power values.

Note

Maximum likelihood (ML) estimates the parameters that maximize the likelihood of the observed data and has the advantage of using all the data and accounting for non-independence (Lynch and Walsh 1998, p. 779; Bolker et al. 2009). On the other hand, ML has the disadvantage of assuming that all fixed effects are known without error, producing a downward bias in the estimation of the residual variance component. This bias can be large if there are lots of fixed effects, especially if

sample sizes are small. Restricted maximum likelihood (REML) has the advantage of not assuming the fixed effects are known and averages over the uncertainty, so there can be less bias in the estimation of the residual variance component. However, REML only maximizes a portion of the likelihood to estimate the effect parameters, but is the preferred method for analyzing large data sets with complex structure.

References

Bolker BM. 2008. Ecological models and data in R. Princeton University Press, New Jersey.

Bolker BM, Brooks ME, Clark CJ, Geange SW, Poulsen JR, Stevens MHH, White J-SS. 2009. Generalized linear mixed models: a practical guide for ecology and evolution. Trends in Ecology and Evolution 24(3): 127-135. DOI: 10.1016/j.tree.2008.10.008

Lynch M, Walsh B. 1998. Genetics and Analysis of Quantitative Traits. Sinauer Associates, Massachusetts.

See Also

powerLmer, powerLmer3

Examples

```
#100 simulations
#pwr_L2<- powerLmer2(varcomp=c(0.19,0.03,0.02,0.66,0.1),nval=c(10,10,20,10),position=2)
#pwr_L2
#5 simulations
pwr_L2<- powerLmer2(varcomp=c(0.19,0.03,0.02,0.66,0.1),nval=c(10,10,20,10),position=2,
nsim=5)
pwr_L2
```

---

powerLmer3 *Power analysis for normal data 3*

Description

Extracts the power values of dam, sire, and dam by sire variance components from a linear mixed-effect model using the *lmer* function of the *lme4* package. Model can include additional fixed and/or random effects.

Usage

```
powerLmer3(var_rand, n_rand, design, remain, var_fix = NULL, n_fix = NULL,
alpha = 0.05, nsim = 100, ml = F, ftest = "LR", iter = NULL)
```

Arguments

var_rand	Vector of known dam, sire, dam by sire, residual, and remaining random variance components, i.e. c(dam,sire,ds,res,rand1,rand2,...).
n_rand	Vector of known dam, sire, family, and remaining random sample sizes, i.e. c(dam,sire,family,rand1,rand2,...).

design	A data frame of the experimental design, using only integers. First three columns must contain and be named "dam", "sire", "family". Remaining columns are the random effects followed by the fixed effects. Continuous fixed effects are a column containing the values 1:nrow(design).
remain	Remaining formula using *lme4* package format. Must be random effects followed by fixed effects.No interactions or random slopes; formulate as intercepts in design.
var_fix	Vector of known fixed variance components, i.e. c(fix1,fix2,...). Continous fixed random values are sorted to match column values.
n_fix	Vector of known fixed sample sizes, i.e. c(fix1,fix2,...). Continuous fixed effects must have a sample size of 1.
alpha	Statistical significance value. Default is 0.05.
nsim	Number of simulations. Default is 100.
ml	Default is FALSE for restricted maximum likelihood. Change to TRUE for maximum likelihood.
ftest	Default is "LR" for likelihood ratio test for fixed effects. Option "PB" is for parametric bootstrap.
iter	Number of iterations for computing the parametric bootstrap significance value for any fixed effects.

Details

Extracts the dam, sire, dam, dam by sire, and any remaining random and fixed effects power values. Power values are calculated by stochastically simulation data and then calculating the proportion of significance values less than *alpha* for each component (Bolker 2008). Significance values for the random effects are determined using likelihood ratio tests (Bolker et al. 2009). Significance values for any fixed effects are determined using likelihood ratio tests or parametric bootstrap method (Bolker et al. 2009) from the *mixed* function of the *afex* package.

Value

A data frame with the sample sizes, variance component inputs, variance component outputs, and power values.

Note

Maximum likelihood (ML) estimates the parameters that maximize the likelihood of the observed data and has the advantage of using all the data and accounting for non-independence (Lynch and Walsh 1998, p. 779; Bolker et al. 2009). On the other hand, ML has the disadvantage of assuming that all fixed effects are known without error, producing a downward bias in the estimation of the residual variance component. This bias can be large if there are lots of fixed effects, especially if sample sizes are small. Restricted maximum likelihood (REML) has the advantage of not assuming the fixed effects are known and averages over the uncertainty, so there can be less bias in the estimation of the residual variance component. However, REML only maximizes a portion of the likelihood to estimate the effect parameters, but is the preferred method for analyzing large data sets with complex structure.

References

Bolker BM. 2008. Ecological models and data in R. Princeton University Press, New Jersey.

Bolker BM, Brooks ME, Clark CJ, Geange SW, Poulsen JR, Stevens MHH, White J-SS. 2009. Generalized linear mixed models: a practical guide for ecology and evolution. Trends in Ecology and Evolution 24(3): 127-135. DOI: 10.1016/j.tree.2008.10.008

Lynch M, Walsh B. 1998. Genetics and Analysis of Quantitative Traits. Sinauer Associates, Massachusetts.

See Also

powerLmer, powerLmer2

Examples

```
##design object: 2 remaining random effects and 1 continous fixed effect
block=c(2,2); blocN=10; position=2; posN=10; offN=20
dam0<- stack(as.data.frame(matrix(1:(block[1]*blocN),ncol=blocN,nrow=block[1])))
sire0<- stack(as.data.frame(matrix(1:(block[2]*blocN),ncol=blocN,nrow=block[2])))
observ0<- merge(dam0,sire0, by="ind")
levels(observ0[,1])<- 1:blocN; colnames(observ0)<- c("block","dam","sire")
observ0$family<- 1:nrow(observ0)  #add family
#expand for position
observ1<- do.call("rbind", replicate(position,observ0,simplify=FALSE));rm(observ0)
observ1$position<- sample(rep(1:posN,each=position)) #random assignment
#expand for offspring
observ<- do.call("rbind", replicate(offN,observ1,simplify=FALSE)); rm(observ1)
desn<- observ[,c(2,3,4,5,1)];rm(observ) #dam,sire,family,position,block
desn$egg_size<- 1:nrow(desn)
colnames(desn)[6]<- "egg_size"

#100 simulations
#pwr_L3<- powerLmer3(var_rand=c(0.19,0.03,0.02,0.51,0.1,0.05),n_rand=c(20,20,40,10,10),
#var_fix=0.1,n_fix=1,design=desn,remain="(1|position)+(1|block)+egg_size")
#pwr_L3
#5 simulations
pwr_L3<- powerLmer3(var_rand=c(0.19,0.03,0.02,0.51,0.1,0.05),n_rand=c(20,20,40,10,10),
var_fix=0.1,n_fix=1,design=desn,remain="(1|position)+(1|block)+egg_size",nsim=5)
pwr_L3
```

---

resampFamily *Bootstrap resample within families*

Description

Bootstrap resample observations grouped by family identities for a specified number of iterations to create a resampled data set.

Usage

```
resampFamily(dat, copy, family, iter)
```

Arguments

`dat`	Data frame observed data to resample.
`copy`	Column numbers to copy.
`family`	Column name containing family identity information.
`iter`	Number of iterations for resampling.

Details

The resampled data can be used for producing bootstrap confidence intervals.

Value

Because of the large file sizes that can be produced, the resampling of each family X is saved separately as a common separated (X_resampF.csv) file in the working directory. These files are merged to create the final resampled data set (resamp_datF.csv).

See Also

`resampRepli`

Examples

```
data(chinook_length) #Chinook salmon offspring length
#resampFamily(dat=chinook_length,copy=c(3:8),family="family",iter=1000)
resampFamily(dat=chinook_length,copy=c(3:8),family="family",iter=2)
#example with a couple iterations
```

---

resampGlmer — *Bootstrap components for non-normal data*

Description

Extracts additive genetic, non-additive genetic, and maternal variance components from a generalized linear mixed-effect model using the *glmer* function of the *lme4* package. Model random effects are dam, sire, and dam by sire.

Usage

```
resampGlmer(resamp, dam, sire, response, fam_link, start, end, quasi = F)
```

Arguments

`resamp`	Data frame of bootstrap resampled data.
`dam`	Column name containing dam(female) parent identity information.
`sire`	Column name containing sire(male) parent identity information.
`response`	Column name containing the offspring (response) phenotype values.
`fam_link`	The family and link in family(link) format. Supported options are binomial(logit), binomial(probit), poisson(log), and poisson (sqrt).
`start`	Starting model number.
`end`	Ending model number.
`quasi`	Incorporate overdispersion or quasi-error structure.

Details

Used for bootstrap resampled data set produced using *resampRepli* or *resampFamily*. Laplace approximation parameter estimation is used, which is a true likelihood method (Bolker et al. 2009). For the overdispersion option, an observation-level random effect is added to the model (Atkins et al. 2013). Extracts the dam, sire, dam, and dam by sire variance components. The residual variance component for the *fam_links* are described by Nakagawa and Schielzeth (2010, 2013). Calculates the total variance component. Calculates the additive genetic, non-additive genetic, and maternal variance components (see Lynch and Walsh 1998, p. 603).

Value

A data frame with columns containing the raw variance components for dam, sire, dam by sire, residual, total, additive genetic, non-additive genetic, and maternal. The number of rows in the data frame matches the number of iterations in the resampled data set and each row represents a model number.

Note

The Laplace approximation is used because there were fewer disadvantages relative to penalized quasi-likelihood and Gauss-Hermite quadrature parameter estimation (Bolker et al. 2009). That is, penalized quasi-likelihood is not recommended for count responses with means less than 5 and binary responses with less than 5 successes per group. Gauss-Hermite quadrature is not recommended for more than two or three random effects because of the rapidly declining analytical speed with the increasing number of random effects.

References

Atkins DC, Baldwin SA, Zheng C, Gallop RJ, Neighbors C. 2013. A tutorial on count regression and zero-altered count models for longitudinal substance use data. Psychology of Addictive Behaviors 27(1): 166-177. DOI: 10.1037/a0029508

Bolker BM, Brooks ME, Clark CJ, Geange SW, Poulsen JR, Stevens MHH, White J-SS. 2009. Generalized linear mixed models: a practical guide for ecology and evolution. Trends in Ecology and Evolution 24(3): 127-135. DOI: 10.1016/j.tree.2008.10.008

Lynch M, Walsh B. 1998. Genetics and Analysis of Quantitative Traits. Sinauer Associates, Massachusetts.

Nakagawa S, Schielzeth H. 2010. Repeatability for Gaussian and non-Gaussian data: a practical guide for biologists. Biological Reviews 85(4): 935-956. DOI: 10.1111/j.1469-185X.2010.00141.x

Nakagawa S, Schielzeth H. 2013. A general and simple method for obtaining R2 from generalized linear mixed-effects models. Methods in Ecology and Evolution 4(2): 133-142. DOI: 10.1111/j.2041-210x.2012.00261.x

See Also

resampGlmer2, resampGlmer3

Examples

```
data(chinook_resampS) #5 iterations

#survival_rcomp<- resampGlmer(resamp=survival_datR,dam="dam",sire="sire",response="status",
#fam_link=binomial(logit),start=1,end=1000)
survival_rcomp<- resampGlmer(resamp=chinook_resampS,dam="dam",sire="sire",response="status",
fam_link=binomial(logit),start=1,end=2)  #first 2 models, a few minutes
```

`resampGlmer2` *Bootstrap components for non-normal data 2*

Description

Extracts additive genetic, non-additive genetic, and maternal variance components from a generalized linear mixed-effect model using the *glmer* function of the *lme4* package. Model random effects are dam, sire, and dam by sire. Options to include one random position and/or one random block effect(s).

Usage

```
resampGlmer2(resamp, dam, sire, response, fam_link, start, end, position = NULL,
block = NULL, quasi = F)
```

Arguments

`resamp`	Data frame of bootstrap resampled data.
`dam`	Column name containing dam(female) parent identity information.
`sire`	Column name containing sire(male) parent identity information.
`response`	Column name containing the offspring (response) phenotype values.
`fam_link`	The family and link in family(link) format. Supported options are binomial(logit), binomial(probit), poisson(log), and poisson (sqrt).
`start`	Starting model number.
`end`	Ending model number.
`position`	Optional column name containing position factor information.
`block`	Optional column name containing block factor information.
`quasi`	Incorporate overdispersion or quasi-error structure.

Details

Used for bootstrap resampled data set produced using *resampRepli* or *resampFamily*. Laplace approximation parameter estimation is used, which is a true likelihood method (Bolker et al. 2009). For the overdispersion option, an observation-level random effect is added to the model (Atkins et al. 2013). Extracts the dam, sire, dam, and dam by sire variance components. Extracts optional position and block variance components. The residual variance component for the *fam_links* are described by Nakagawa and Schielzeth (2010, 2013). Calculates the total variance component. Calculates the additive genetic, non-additive genetic, and maternal variance components (see Lynch and Walsh 1998, p. 603).

Value

A data frame with columns containing the raw variance components for dam, sire, dam by sire, residual, total, additive genetic, non-additive genetic, and maternal. Also columns containing the raw variance components for the options of position and/or block. The number of rows in the data frame matches the number of iterations in the resampled data set and each row represents a model number.

Note

The Laplace approximation is used because there were fewer disadvantages relative to penalized quasi-likelihood and Gauss-Hermite quadrature parameter estimation (Bolker et al. 2009). That is, penalized quasi-likelihood is not recommended for count responses with means less than 5 and binary responses with less than 5 successes per group. Gauss-Hermite quadrature is not recommended for more than two or three random effects because of the rapidly declining analytical speed with the increasing number of random effects.

References

Atkins DC, Baldwin SA, Zheng C, Gallop RJ, Neighbors C. 2013. A tutorial on count regression and zero-altered count models for longitudinal substance use data. Psychology of Addictive Behaviors 27(1): 166-177. DOI: 10.1037/a0029508

Bolker BM, Brooks ME, Clark CJ, Geange SW, Poulsen JR, Stevens MHH, White J-SS. 2009. Generalized linear mixed models: a practical guide for ecology and evolution. Trends in Ecology and Evolution 24(3): 127-135. DOI: 10.1016/j.tree.2008.10.008

Lynch M, Walsh B. 1998. Genetics and Analysis of Quantitative Traits. Sinauer Associates, Massachusetts.

Nakagawa S, Schielzeth H. 2010. Repeatability for Gaussian and non-Gaussian data: a practical guide for biologists. Biological Reviews 85(4): 935-956. DOI: 10.1111/j.1469-185X.2010.00141.x

Nakagawa S, Schielzeth H. 2013. A general and simple method for obtaining R2 from generalized linear mixed-effects models. Methods in Ecology and Evolution 4(2): 133-142. DOI: 10.1111/j.2041-210x.2012.00261.x

See Also

`resampGlmer`, `resampGlmer3`

Examples

```
data(chinook_resampS) #5 iterations

#survival_rcomp2<- resampGlmer2(resamp=survival_datR,dam="dam",sire="sire",response="status",
#fam_link=binomial(logit),start=1,end=1000,position="tray")
survival_rcomp2<- resampGlmer2(resamp=chinook_resampS,dam="dam",sire="sire",response="status",
fam_link=binomial(logit),start=1,end=2,position="tray")  #first 2 models, a few minutes
```

`resampGlmer3` *Bootstrap components for non-normal data 3*

Description

Extracts additive genetic, non-additive genetic, and maternal variance components from a generalized linear mixed-effect model using the *glmer* function of the *lme4* package. Model random effects are dam, sire, dam by sire, and any additional fixed and/or random effects.

Usage

```
resampGlmer3(resamp, dam, sire, response, fam_link, start, end, remain, quasi = F)
```

Arguments

`resamp`	Data frame of bootstrap resampled data.
`dam`	Column name containing dam(female) parent identity information.
`sire`	Column name containing sire(male) parent identity information.
`response`	Column name containing the offspring (response) phenotype values.
`fam_link`	The family and link in family(link) format. Supported options are binomial(logit), binomial(probit), poisson(log), and poisson (sqrt).
`start`	Starting model number.
`end`	Ending model number.
`remain`	Remaining formula using *lme4* package format with # sign (see column names), e.g. fixed# + (1|random#).
`quasi`	Incorporate overdispersion or quasi-error structure.

Details

Used for bootstrap resampled data set produced using *resampRepli* or *resampFamily*. Laplace approximation parameter estimation is used, which is a true likelihood method (Bolker et al. 2009). For the overdispersion option, an observation-level random effect is added to the model (Atkins et al. 2013). Extracts the dam, sire, dam, and dam by sire variance components. Extracts any additional fixed effect and random effect variance components. The fixed-effect variance component is as a single group using the method described by Nakagawa and Schielzeth (2013). The residual variance component for the *fam_links* are described by Nakagawa and Schielzeth (2010, 2013). Calculates the total variance component. Calculates the additive genetic, non-additive genetic, and maternal variance components (see Lynch and Walsh 1998, p. 603).

Value

A data frame with columns containing the raw variance components for dam, sire, dam by sire, residual, total, additive genetic, non-additive genetic, and maternal. Also columns containing the raw variance components for remaining formula components. The number of rows in the data frame matches the number of iterations in the resampled data set and each row represents a model number.

Note

The Laplace approximation is used because there were fewer disadvantages relative to penalized quasi-likelihood and Gauss-Hermite quadrature parameter estimation (Bolker et al. 2009). That is, penalized quasi-likelihood is not recommended for count responses with means less than 5 and binary responses with less than 5 successes per group. Gauss-Hermite quadrature is not recommended for more than two or three random effects because of the rapidly declining analytical speed with the increasing number of random effects.

References

Atkins DC, Baldwin SA, Zheng C, Gallop RJ, Neighbors C. 2013. A tutorial on count regression and zero-altered count models for longitudinal substance use data. Psychology of Addictive Behaviors 27(1): 166-177. DOI: 10.1037/a0029508

Bolker BM, Brooks ME, Clark CJ, Geange SW, Poulsen JR, Stevens MHH, White J-SS. 2009. Generalized linear mixed models: a practical guide for ecology and evolution. Trends in Ecology and Evolution 24(3): 127-135. DOI: 10.1016/j.tree.2008.10.008

Lynch M, Walsh B. 1998. Genetics and Analysis of Quantitative Traits. Sinauer Associates, Massachusetts.

Nakagawa S, Schielzeth H. 2010. Repeatability for Gaussian and non-Gaussian data: a practical guide for biologists. Biological Reviews 85(4): 935-956. DOI: 10.1111/j.1469-185X.2010.00141.x

Nakagawa S, Schielzeth H. 2013. A general and simple method for obtaining R2 from generalized linear mixed-effects models. Methods in Ecology and Evolution 4(2): 133-142. DOI: 10.1111/j.2041-210x.2012.00261.x

See Also

resampGlmer, resampGlmer2

Examples

```
data(chinook_resampS) #5 iterations

#survival_rcomp3<- resampGlmer3(resamp=survival_datR,dam="dam",sire="sire",
#response="status",fam_link=binomial(logit),start=1,end=1000,
#remain="egg_size# + (1|tray#)")
survival_rcomp3<- resampGlmer3(resamp=chinook_resampS,dam="dam",sire="sire",
response="status",fam_link=binomial(logit),start=1,end=2,
remain="egg_size# + (1|tray#)") #first 2 models, a few minutes
```

---

resampLmer — *Bootstrap components for normal data*

Description

Extracts additive genetic, non-additive genetic, and maternal variance components from a linear mixed-effect model using the *lmer* function of the *lme4* package. Model random effects are dam, sire, and dam by sire.

Usage

```
resampLmer(resamp, dam, sire, response, start, end, ml = F)
```

Arguments

resamp	Data frame of bootstrap resampled data.
dam	Column name containing dam(female) parent identity information.
sire	Column name containing sire(male) parent identity information.
response	Column name containing the offspring (response) phenotype values.
start	Starting model number.
end	Ending model number.
ml	Default is FALSE for restricted maximum likelihood. Change to TRUE for maximum likelihood.

Details

Used for bootstrap resampled data set produced using *resampRepli* or *resampFamily*. Extracts the dam, sire, dam, dam by sire, and residual variance components. Calculates the total variance component. Calculates the additive genetic, non-additive genetic, and maternal variance components (see Lynch and Walsh 1998, p. 603).

Value

A data frame with columns containing the raw variance components for dam, sire, dam by sire, residual, total, additive genetic, non-additive genetic, and maternal. The number of rows in the data frame matches the number of iterations in the resampled data set and each row represents a model number.

Note

Maximum likelihood (ML) estimates the parameters that maximize the likelihood of the observed data and has the advantage of using all the data and accounting for non-independence (Lynch and Walsh 1998, p. 779; Bolker et al. 2009). On the other hand, ML has the disadvantage of assuming that all fixed effects are known without error, producing a downward bias in the estimation of the residual variance component. This bias can be large if there are lots of fixed effects, especially if sample sizes are small. Restricted maximum likelihood (REML) has the advantage of not assuming the fixed effects are known and averages over the uncertainty, so there can be less bias in the estimation of the residual variance component. However, REML only maximizes a portion of the likelihood to estimate the effect parameters, but is the preferred method for analyzing large data sets with complex structure.

References

Bolker BM, Brooks ME, Clark CJ, Geange SW, Poulsen JR, Stevens MHH, White J-SS. 2009. Generalized linear mixed models: a practical guide for ecology and evolution. Trends in Ecology and Evolution 24(3): 127-135. DOI: 10.1016/j.tree.2008.10.008

Lynch M, Walsh B. 1998. Genetics and Analysis of Quantitative Traits. Sinauer Associates, Massachusetts.

See Also

resampLmer2, resampLmer3

Examples

```
data(chinook_resampL) #5 iterations

#length_rcomp1<- resampLmer(resamp=length_datR,dam="dam",sire="sire",response="length",
#start=1,end=1000)
length_rcomp1<- resampLmer(resamp=chinook_resampL,dam="dam",sire="sire",response="length",
start=1,end=5)
```

resampLmer2 *Bootstrap components for normal data 2*

Description

Extracts additive genetic, non-additive genetic, and maternal variance components from a linear mixed-effect model using the *lmer* function of the *lme4* package. Model random effects are dam, sire, and dam by sire. Options to include one random position and/or one random block effect(s).

Usage

```
resampLmer2(resamp, dam, sire, response, start, end, position = NULL, block = NULL,
ml = F)
```

Arguments

resamp	Data frame of bootstrap resampled data.
dam	Column name containing dam(female) parent identity information.
sire	Column name containing sire(male) parent identity information.
response	Column name containing the offspring (response) phenotype values.
start	Starting model number.
end	Ending model number.
position	Optional column name containing position factor information.
block	Optional column name containing block factor information.
ml	Default is FALSE for restricted maximum likelihood. Change to TRUE for maximum likelihood.

Details

Used for bootstrap resampled data set produced using *resampRepli* or *resampFamily*. Extracts the dam, sire, dam, dam by sire, and residual variance components. Extracts optional position and block variance components. Calculates the total variance component. Calculates the additive genetic, non-additive genetic, and maternal variance components (see Lynch and Walsh 1998, p. 603).

Value

A data frame with columns containing the raw variance components for dam, sire, dam by sire, residual, total, additive genetic, non-additive genetic, and maternal. Also columns containing the raw variance components for the options of position and/or block. The number of rows in the data frame matches the number of iterations in the resampled data set and each row represents a model number.

Note

Maximum likelihood (ML) estimates the parameters that maximize the likelihood of the observed data and has the advantage of using all the data and accounting for non-independence (Lynch and Walsh 1998, p. 779; Bolker et al. 2009). On the other hand, ML has the disadvantage of assuming that all fixed effects are known without error, producing a downward bias in the estimation of the residual variance component. This bias can be large if there are lots of fixed effects, especially if

sample sizes are small. Restricted maximum likelihood (REML) has the advantage of not assuming the fixed effects are known and averages over the uncertainty, so there can be less bias in the estimation of the residual variance component. However, REML only maximizes a portion of the likelihood to estimate the effect parameters, but is the preferred method for analyzing large data sets with complex structure.

References

Bolker BM, Brooks ME, Clark CJ, Geange SW, Poulsen JR, Stevens MHH, White J-SS. 2009. Generalized linear mixed models: a practical guide for ecology and evolution. Trends in Ecology and Evolution 24(3): 127-135. DOI: 10.1016/j.tree.2008.10.008

Lynch M, Walsh B. 1998. Genetics and Analysis of Quantitative Traits. Sinauer Associates, Massachusetts.

See Also

resampLmer, resampLmer3

Examples

```
data(chinook_resampL) #5 iterations

#length_rcomp2<- resampLmer2(resamp=length_datR,dam="dam",sire="sire",response="length",
#start=1,end=1000,position="tray")
length_rcomp2<- resampLmer2(resamp=chinook_resampL,dam="dam",sire="sire",response="length",
start=1,end=5,position="tray")
```

---

resampLmer3 *Bootstrap components for normal data 3*

Description

Extracts additive genetic, non-additive genetic, and maternal variance components from a linear mixed-effect model using the *lmer* function of the *lme4* package. Model random effects are dam, sire, dam by sire, and any additional fixed and/or random effects.

Usage

```
resampLmer3(resamp, dam, sire, response, start, end, remain, ml = F)
```

Arguments

resamp	Data frame of bootstrap resampled data.
dam	Column name containing dam(female) parent identity information.
sire	Column name containing sire(male) parent identity information.
response	Column name containing the offspring (response) phenotype values.
start	Starting model number.
end	Ending model number.
remain	Remaining formula using *lme4* package format with # sign (see column names), e.g. fixed# + (1|random#).
ml	Default is FALSE for restricted maximum likelihood. Change to TRUE for maximum likelihood.

Details

Used for bootstrap resampled data set produced using *resampRepli* or *resampFamily*. Extracts the dam, sire, dam, dam by sire, and residual variance components. Extracts any additional fixed effect and random effect variance components. The fixed-effect variance component is as a single group using the method described by Nakagawa and Schielzeth (2013). Calculates the total variance component. Calculates the additive genetic, non-additive genetic, and maternal variance components (see Lynch and Walsh 1998, p. 603).

Value

A data frame with columns containing the raw variance components for dam, sire, dam by sire, residual, total, additive genetic, non-additive genetic, and maternal. Also columns containing the raw variance components for remaining formula components. The number of rows in the data frame matches the number of iterations in the resampled data set and each row represents a model number.

Note

Maximum likelihood (ML) estimates the parameters that maximize the likelihood of the observed data and has the advantage of using all the data and accounting for non-independence (Lynch and Walsh 1998, p. 779; Bolker et al. 2009). On the other hand, ML has the disadvantage of assuming that all fixed effects are known without error, producing a downward bias in the estimation of the residual variance component. This bias can be large if there are lots of fixed effects, especially if sample sizes are small. Restricted maximum likelihood (REML) has the advantage of not assuming the fixed effects are known and averages over the uncertainty, so there can be less bias in the estimation of the residual variance component. However, REML only maximizes a portion of the likelihood to estimate the effect parameters, but is the preferred method for analyzing large data sets with complex structure.

References

Bolker BM, Brooks ME, Clark CJ, Geange SW, Poulsen JR, Stevens MHH, White J-SS. 2009. Generalized linear mixed models: a practical guide for ecology and evolution. Trends in Ecology and Evolution 24(3): 127-135. DOI: 10.1016/j.tree.2008.10.008

Lynch M, Walsh B. 1998. Genetics and Analysis of Quantitative Traits. Sinauer Associates, Massachusetts.

Nakagawa S, Schielzeth H. 2013. A general and simple method for obtaining R2 from generalized linear mixed-effects models. Methods in Ecology and Evolution 4(2): 133-142. DOI: 10.1111/j.2041-210x.2012.00261.x

See Also

resampLmer, resampLmer2

Examples

```
data(chinook_resampL)

#length_rcomp3<- resampLmer3(resamp=length_datR,dam="dam",sire="sire",response="length",
#start=1,end=1000,remain="egg_size# + (1|tray#)")
length_rcomp3<- resampLmer3(resamp=chinook_resampL,dam="dam",sire="sire",response="length",
start=1,end=5,remain="egg_size# + (1|tray#)")
```

resampRepli *Bootstrap resample within replicates*

Description

Bootstrap resample observations grouped by replicate identities within family identities for a specified number of iterations to create a resampled data set.

Usage

```
resampRepli(dat, copy, family, replicate, iter)
```

Arguments

dat	Data frame observed data to resample.
copy	Column numbers to copy.
family	Column name containing family identity information.
replicate	Column name containing replicate identity information.
iter	Number of iterations for resampling.

Details

The resampled data can be used for producing bootstrap confidence intervals.

Value

Because of the large file sizes that can be produced, the resampling of each replicate Y per family X is saved separately as a common separated (X_Y_resampR.csv) file in the working directory. These files are merged to create the final resampled data set (resamp_datR.csv).

See Also

resampFamily

Examples

```
data(chinook_length) #Chinook salmon offspring length
#resampRepli(dat=chinook_length,copy=c(3:8),family="family",replicate="repli",iter=1000)
resampRepli(dat=chinook_length,copy=c(3:8),family="family",replicate="repli",iter=2)
#example with a couple iterations
```

Index

∗Topic **datasets**
chinook_bootL, 10
chinook_bootS, 11
chinook_jackL, 12
chinook_length, 13
chinook_resampL, 14
chinook_resampS, 15
chinook_survival, 16
∗Topic **package**
fullfact-package, 2

barMANA, 5
boxMANA, 6
buildBinary, 8, *9*
buildMulti, *8*, 9

chinook_bootL, 10
chinook_bootS, 11
chinook_jackL, 12
chinook_length, 13
chinook_resampL, 14
chinook_resampS, 15
chinook_survival, 16
ciJack, 17, *20*, *21*
ciJack2, *18*, 19, *21*
ciJack3, *18*, *20*, 20
ciMANA, 21, *23*, *25*
ciMANA2, *22*, 22, *25*
ciMANA3, *22*, *23*, 24

fullfact (fullfact-package), 2
fullfact-package, 2

JackGlmer, 25, *29*, *31*
JackGlmer2, *27*, 27, *31*
JackGlmer3, *27*, *29*, 29
JackLmer, 31, *34*, *36*
JackLmer2, *33*, 33, *36*
JackLmer3, *33*, *34*, 35

observGlmer, 37, *39*, *41*
observGlmer2, *38*, 38, *41*
observGlmer3, *38*, *39*, 40
observLmer, 42, *44*, *46*
observLmer2, *43*, 43, *46*
observLmer3, *43*, *44*, 44

powerGlmer, 46, *49*, *50*
powerGlmer2, *47*, 47, *50*
powerGlmer3, *47*, *49*, 49
powerLmer, 51, *54*, *56*
powerLmer2, *52*, 53, *56*
powerLmer3, *52*, *54*, 54

resampFamily, 56, *67*
resampGlmer, 57, *60*, *62*
resampGlmer2, *58*, 59, *62*
resampGlmer3, *58*, *60*, 60
resampLmer, 62, *65*, *66*
resampLmer2, *63*, 64, *66*
resampLmer3, *63*, *65*, 65
resampRepli, *57*, 67
