# Supplementary material for: fullfact: an R package for the analysis of genetic and maternal variance components from full factorial mating designs
Source: Ecol Evol. 2016 Feb 14;6(6):1656–65. doi: 10.1002/ece3.1943 (PMC4752957; doi:10.1002/ece3.1943)
Supplement: Supplementary file 10 [file ECE3-6-1656-s010.docx]

Supplementary Information for: “fullfact: an R package for the analysis of genetic and maternal variance components from full factorial mating designs”

Supplementary Information S1. Additional models from Chinook salmon survival to hatching worked example described in main text.

Supplementary Information S2. Simulated data example of survival.

Supplementary Information S3. Worked example for Chinook salmon length at hatch.

Supplementary Information S1. Additional models from Chinook salmon survival to hatching worked example described in main text.

Here, we provide two additional models from the worked example of Chinook salmon survival described in the main text. We also provide a model of Chinook salmon length using replicate-level data (i.e. family mean per replicate) to illustrate the change in variance component estimates relative to individual-level data described in the main text and the supplementary information below. The survival and length data were from an 11 × 11 factorial breeding design which was originally analyzed by Pitcher & Neff (2007).

**Reduced model for Chinook salmon survival to hatching using binary data**

For reducing the model using the observGlmer function, the input parameters are similar to the full model function, with the exception of no ‘remain’ parameter.

survival_mod2<- observGlmer(observ=chinook_survival2,dam="dam",sire="sire", response="status",fam_link=binomial(logit))

survival_mod2

$random

effect variance percent d.AIC d.BIC Chi.sq p.value

1 dam:sire 0.1673003 3.788025 636.18699 627.68742 638.18699 8.284084e-141

2 sire 0.1663766 3.767111 44.34824 35.84867 46.34824 9.899641e-12

3 dam 0.7930131 17.955454 145.47110 136.97153 147.47110 6.190795e-34

$other

component variance percent

1 Residual 3.289868 74.48941

2 Total 4.416558 100.00000

$calculation

component variance percent

1 additive 0.6655065 15.06844

2 nonadd 0.6692012 15.15210

3 maternal 0.6266364 14.18834

The output displays similar results to the full model. The significant random effects are dam (18.0% of the phenotypic variance), sire (3.8%), and dam by sire (3.8%). Additive genetic, non-additive genetic, and maternal variance explain similar amounts of the phenotypic variance (15.1, 15.2, and 14.2%).

**Test for overdispersion for Chinook salmon survival to hatching using proportion data**

repli_mod1<- observGlmer(observ=chinook_survival,dam="dam",sire="sire", response="proportion",fam_link=binomial(logit),quasi=T)

repli_mod1

$random

effect variance percent d.AIC d.BIC Chi.sq p.value

1 dispersion 0.0000000 0.000000 -1.9999999 -5.4889376 8.325375e-08 0.99976978

2 dam:sire 0.0000000 0.000000 -2.0000000 -5.4889377 1.223896e-09 0.99997209

3 sire 0.1625763 4.110349 -0.5554632 -4.0444009 1.444537e+00 0.22940659

4 dam 0.5028487 12.713311 3.8489369 0.3599992 5.848937e+00 0.01558646

$other

component variance percent

1 Residual 3.289868 83.17634

2 Total 3.955293 100.00000

$calculation

component variance percent

1 additive 0.6503054 16.441395

2 nonadd 0.0000000 0.000000

3 maternal 0.3402724 8.602962

The dispersion random effect is non-significant, thus it can be removed from the model.

**Analysis of Chinook salmon length at hatch using family mean per replicate data**

repli_mod2<- observLmer2(observ=chinook_lengthF,dam="dam",sire="sire", response="length",position="tray")

repli_mod2

$random

effect variance percent d.AIC d.BIC Chi.sq p.value

1 dam:sire 0.02760088 3.777174 -1.571675 -5.060612 0.4283253 5.128118e-01

2 tray 0.08721164 11.934896 14.675568 11.186630 16.6755682 4.434850e-05

3 sire 0.00000000 0.000000 -2.000000 -5.488938 0.0000000 1.000000e+00

4 dam 0.20149112 27.574021 30.379053 26.890115 32.3790529 1.268465e-08

$other

component variance percent

1 Residual 0.4144245 56.71391

2 Total 0.7307281 100.00000

$calculation

component variance percent

1 additive 0.0000000 0.00000

2 nonadd 0.1104035 15.10870

3 maternal 0.2014911 27.57402

Compared to the individual-level data, non-additive genetic variance decreased (new value of 15.1%), maternal variance increased (27.6%), and there was no change in the amount of additive genetic variance (0%). The individual-level data estimates were 0% additive genetic variance, 69.1% non-additive genetic variance, and 19.5% maternal variance (see worked example below in supplementary information S3).

**References**

Pitcher, T.E. & Neff, B.D. (2007). Genetic quality and offspring performance in Chinook salmon: Implications for supportive breeding. *Conservation Genetics*, **8**, 607–616.

Supplementary Information S2. Simulated data example of survival.

In the main text, we went through the entire workflow for empirical data of survival of unknown variance components. Here, we simulate binary data for survival using known variance components and to illustrate the accuracy and precision of the confidence intervals. Data are simulated with actual variance components that explain 15.2% additive genetic (4 × 0.176 / 4.628), 3.6% non-additive genetic, and 20.4% maternal of the phenotypic variance. The simulation used 10 dams and sires with 400 offspring per family.

nval<- c(10,10,400) #10 dams and sires, 400 offspring

resK<- (pi^2)/3 #residual variance binomial(logit)

damN<- nval[1];sireN<- nval[2];offN<- nval[3]

#variance components

damR<- rnorm(damN,0,sd=sqrt(1.17)); var(damR) #actual 1.120853

sireR<- rnorm(sireN,0,sd=sqrt(0.19)) #0.1761597

dxsR<- rnorm(damN*sireN,0,sd=sqrt(0.05)) #0.04147418

# total= 4.628355= 1.120853+ 0.1761597+ 0.04147418+ (pi^2)/3

#create data frame

observ <- expand.grid(off=1:offN, dam=1:damN, sire=1:sireN)

observ$famil<- rep(1:(damN*sireN),each=offN)

#add binary response variable

observ<- within(observ, { resp<- damR[dam] + sireR[sire] + dxsR[famil] } )

observ<- within(observ, { status<- rbinom(nrow(observ),prob=plogis(resp), size=1)})

We use the observGlmer function to estimate the variance components of the simulated data. Bootstrap-*t* confidence intervals for the estimated additive genetic, non-additive genetic, and maternal variance components are produced by first resampling the data using the resampFamily function, then applying models to the resampled data using the resampGlmer function, and extracting the 95% interval using ciMANA.

#estimate variance components

survival_sim<- observGlmer(observ=observ, dam="dam", sire="sire", response= "status", fam_link=binomial(logit), ml=F)

survival_sim

$random

effect variance percent d.AIC d.BIC Chi.sq p.value

1 dam:sire 0.03500267 0.7634198 114.6476 106.05096 116.6476 3.428872e-27

2 sire 0.18325850 3.9969282 105.9314 97.33479 107.9314 2.782412e-25

3 dam 1.07685434 23.4865469 237.0613 228.46466 239.0613 6.300666e-54

$other

component variance percent

1 Residual 3.289868 71.75311

2 Total 4.584984 100.00000

$calculation

component variance percent

1 additive 0.7330340 15.987713

2 nonadd 0.1400107 3.053679

3 maternal 0.8935958 19.489619

#resample

resampFamily(dat=observ, copy=c(2,3,6), family="famil", iter=1000)

#models

survival_dat<- read.csv("resamp_datF.csv") #import file from directory

survival_rcomp<- resampGlmer(resamp=survival_dat, dam="dam", sire="sire", response="status", fam_link=binomial(logit), start=1, end=1000)

#extract confidence intervals

ciMANA(comp=survival_rcomp, level=95)

$raw

component lower median upper

1 additive 0.633 0.715 0.806

2 nonadd 0.157 0.205 0.258

3 maternal 0.850 0.900 0.956

$percentage

component lower median upper

1 additive 13.8 15.6 17.4

2 nonadd 3.4 4.5 5.6

3 maternal 18.6 19.6 20.6

The 95% confidence intervals for the additive genetic, non-additive genetic, and maternal variance components contain the actual values of the simulated data.

Supplementary Information S3. Worked example for Chinook salmon length at hatch.

In the main text, we went through the entire workflow for a binary variable (i.e. survival). Also for the 11 × 11 factorial mating of Chinook salmon described in the main text the total body length (nearest 0.1 mm) at hatch of five haphazardly selected individuals was collected for each of the two replicates (total *n* = 10 individuals per family) (Pitcher & Neff 2007). Here, we go through the entire workflow for a continuous variable (i.e. length), provide an example of bias and acceleration correction of bootstrap-*t* confidence intervals, and provide an example of jackknife confidence intervals.

*Step 1: Analysis of variance components*

The length data of the Chinook salmon individuals were confirmed visually for approximate normality using a histogram. Similar to the survival to hatching example (see main text), we previously used the observGlmer3 function to test the fixed effect of egg size and random position effects of tray and cell.

length_mod1<- observLmer3(observ=chinook_length,dam="dam",sire="sire", response="length",remain="egg_size + (1|tray) + (1|cell)")

length_mod1

$fixed

effect variance percent Chi.sq p.value

1 egg_size NA NA 3.2527 0.1209

2 Fix_Tot 0.05927422 5.62306 NA NA

$LRT.fixed

term d.AIC d.BIC Chi.sq p.value

1 egg_size 1.252684 -3.845691 3.252684 0.07130659

$random

effect effect2 variance percent

1 dam:sire (Intercept) 0.176762897 16.768645

2 cell (Intercept) 0.001996391 0.189388

3 tray (Intercept) 0.119846599 11.369270

4 sire (Intercept) 0.000000000 0.000000

5 dam (Intercept) 0.146355276 13.884021

$LRT.random

term d.AIC d.BIC Chi.sq p.value

1 (1 | dam) 14.181458 9.083083 1.618146e+01 5.755471e-05

2 (1 | sire) -2.000000 -7.098376 3.001333e-11 9.999956e-01

3 (1 | dam:sire) 120.877444 115.779068 1.228774e+02 1.483341e-28

4 (1 | tray) 122.917829 117.819453 1.249178e+02 5.304644e-29

5 (1 | cell) -1.973175 -7.071551 2.682450e-02 8.699029e-01

$other

component variance percent

1 Residual 0.5498921 52.16562

2 Total 1.0541275 100.00000

$calculation

component variance percent

1 additive 0.0000000 0.00000

2 nonadd 0.7070516 67.07458

3 maternal 0.1463553 13.88402

The output object contains the fixed effect of egg size which is non-significant, but there is a trend (*p* = 0.12) that explains 5.6% of the phenotypic variance. The significant random effects are tray position (11.4% of the phenotypic variance), dam (13.9%), and dam by sire (16.8%). Non-significant effects are sire (0%) and cell position (0.2%). Non-additive genetic variance explains the most phenotypic variance (67.1%), followed by maternal variance (13.9%), and there is no additive genetic variance (0%). There is only one significant random position effect (i.e. tray) and the fixed effect (i.e. egg size) is statistically non-significant, so we can reduce the number of effects and use the observLmer2 function to make a new model object (length_mod2) because it can handle one random position effect. The input parameters are similar to the previous function, with the exception of no ‘remain’ parameter and the column name for the random position effect is in quotations.

length_mod2<- observLmer2(observ=chinook_length,dam="dam",sire="sire", response="length",position="tray")

length_mod2

$random

effect variance percent d.AIC d.BIC Chi.sq p.value

1 dam:sire 0.1798063 17.28172 141.49865 136.400272 1.434986e+02 4.573042e-33

2 tray 0.1077422 10.35543 121.50329 116.404911 1.235033e+02 1.082069e-28

3 sire 0.0000000 0.00000 -2.00000 -7.098376 1.818989e-12 9.999989e-01

4 dam 0.2029683 19.50788 29.98924 24.890867 3.198924e+01 1.550287e-08

$other

component variance percent

1 Residual 0.5499255 52.85497

2 Total 1.0404423 100.00000

$calculation

component variance percent

1 additive 0.0000000 0.00000

2 nonadd 0.7192252 69.12687

3 maternal 0.2029683 19.50788

The output object shows that the values for tray, dam by sire, additive genetic, and non-additive genetic changed minimally (< 2%) from the previous model, whereas the values for both dam and maternal increased by 5.6%, the same value for egg size in the previous model. This later result suggests that properties associated with egg size could explain around a little over a quarter (28.7% = 5.6 / 19.5%) of the dam and maternal variance. Although egg size was statistically non-significant in the previous model, it may be considered biologically significant and could potentially be kept in the model as a fixed effect.

*Step 2: Production of confidence intervals for variance components*

Similar to the survival to hatching example (see main text), first the observed data for every replicate within family is bootstrap resampled with using the resampRepli function. The input contains the observed data frame (i.e. chinook_length) and the column numbers to copy as a vector, so we include the column numbers for response (i.e. length, 7), dam (3), and sire (4). We also include the column number for tray (5) because this random position effect was significant for the observed data.

resampRepli(dat=chinook_length,copy=c(3,4,5,7),family="family",replicate="repli",iter=1000)

Second, we use the resampLmer2 function to apply a common model to each iteration of the bootstrap resampled data. The input also contains the column name for the random position effect.

chinook_resampL<- read.csv("length_datR.csv") #resamp data, working directory

chinook_bootL<- resampLmer2(resamp=chinook_resampL,dam="dam",sire="sire", response="length",start=1,end=1000,position="tray")

head(chinook_bootL)

dam.sire tray sire dam Residual Total additive nonadd maternal

1 0.2164484 0.11224115 0.00e+00 0.1892240 0.4954401 1.013354 0.00e+00 0.8657934 0.1892240

2 0.2169366 0.11793698 0.00e+00 0.2018556 0.5453407 1.082070 0.00e+00 0.8677465 0.2018556

3 0.2088344 0.09537758 3.59e-16 0.1875733 0.5281606 1.019946 1.44e-15 0.8353375 0.1875733

4 0.1959661 0.12697427 0.00e+00 0.1802369 0.5212489 1.024426 0.00e+00 0.7838645 0.1802369

5 0.2025702 0.12455151 0.00e+00 0.2005369 0.5110783 1.038737 0.00e+00 0.8102810 0.2005369

6 0.2302385 0.09859192 3.17e-15 0.1900782 0.5352927 1.054201 1.27e-14 0.9209539 0.1900782

Third, we extract the raw bootstrap-*t* confidence intervals and median from the variance component data frame using the ciMANA2 function.

length_ci1<- ciMANA2(comp=chinook_bootL,level=95,position="tray")

length_ci1

$raw

component lower median upper

1 additive 0.000 0.000 0.031

2 nonadd 0.716 0.847 0.994

3 maternal 0.167 0.200 0.233

4 tray 0.092 0.113 0.135

$percentage

component lower median upper

1 additive 0.0 0.0 3.0

2 nonadd 69.0 81.0 94.8

3 maternal 16.6 19.2 21.9

4 tray 8.9 10.8 12.9

The median for the non-additive genetic variance component (81.0%) is not close to the observed value (69.1%), suggesting we should try a bias and acceleration correction for the bootstrap-*t* confidence intervals. To calculate the acceleration value for the correction, we need to produce a jackknife resample of the observed data. The JackLmer2 function is used for delete-one jackknife resampling of the observed data (i.e. chinook_jackL). This time using the ciMANA2 function again we include the ‘bias’ input containing the raw observed values of the variance components, i.e. additive, non-additive, maternal, and position (in this order). We also include the ‘accel’ input containing the jackknife data set.

chinook_jackL<- JackLmer2(observ=chinook_length,dam="dam",sire="sire", response="length",position="tray")

head(chinook_jackL)

dam.sire tray sire dam Residual Total additive nonadd maternal

1 0.1806919 0.1077726 0.000000e+00 0.2047354 0.5499255 1.043125 0.000000e+00 0.7227677 0.2047354

2 0.1798059 0.1077512 0.000000e+00 0.2030566 0.5499255 1.040539 0.000000e+00 0.7192234 0.2030566

3 0.1791295 0.1077520 8.417216e-15 0.2043530 0.5499255 1.041160 3.366887e-14 0.7165178 0.2043530

4 0.1798260 0.1077423 0.000000e+00 0.2029309 0.5499255 1.040425 0.000000e+00 0.7193039 0.2029309

5 0.1794712 0.1077996 0.000000e+00 0.2021526 0.5499255 1.039349 0.000000e+00 0.7178846 0.2021526

6 0.1798302 0.1077523 0.000000e+00 0.2027353 0.5499255 1.040243 0.000000e+00 0.7193206 0.2027353

length_ci2<- ciMANA2(comp=chinook_bootL,level=95,position="tray",bias=c( 0.0000000,0.7192252,0.2029683,1.0404423,0.1077422),accel=chinook_jackL)

length_ci2

$raw

component lower median upper change

1 additive 0.000 0.000 0.031 bias fail

2 nonadd 0.598 0.601 0.725 <NA>

3 maternal 0.172 0.206 0.240 <NA>

4 tray 0.084 0.103 0.127 <NA>

$percentage

component lower median upper change

1 additive 0.0 0.0 3.0 bias fail

2 nonadd 57.8 58.4 70.6 <NA>

3 maternal 17.0 19.7 22.4 <NA>

4 tray 8.3 10.0 12.0 <NA>

The median for the non-additive genetic variance component is lower (58.4%) but is still not close to the observed value (69.1%). The ‘bias fail’ warning is because of the zero observed value for the additive genetic variance component causing a bias calculation of infinity, so the uncorrected confidence interval is presented for this component. Another option is calculate confidence intervals directly from the jackknife data set using the ciJack2 function. The inputs are the jackknife data set and the observed values in ‘full’.

length_ci3<- ciJack2(comp=chinook_jackL,level=95,position="tray",full=c( 0.0000000,0.7192252,0.2029683,1.0404423,0.1077422))

length_ci3

$raw

component lower mean upper

1 additive 0.000 0.000 0.000

2 nonadd 0.511 0.721 0.931

3 maternal 0.159 0.206 0.253

4 tray 0.075 0.107 0.139

$percentage

component lower mean upper

1 additive 0.0 0.0 0.0

2 nonadd 51.8 69.3 86.7

3 maternal 15.9 19.7 23.6

4 tray 7.6 10.3 13.0

*Step 3: Visualization of the confidence intervals*

A simple plot of the additive genetic, non-additive genetic, and maternal 95% confidence intervals and means from the jackknife method is produced using the barMANA function (Figure S1). The input contains the confidence interval object (i.e. length_ci3). After looking at the initial plot (not shown), we included additional input parameters for the error bar length, unit increment of the y-axis, maximum value of the y-axis, and the size of the label on the y-axis. The plot shows that non-additive genetic and maternal effects, and not additive genetic effects, are contributing to the phenotypic variance of length at hatch for the Chinook salmon 11 × 11 factorial mating.

**References**

Pitcher, T.E. & Neff, B.D. (2007). Genetic quality and offspring performance in Chinook salmon: Implications for supportive breeding. *Conservation Genetics*, **8**, 607–616.

barMANA(ci_dat=length_ci3,bar_len=0.3,yunit=20,ymax=100,cex_ylab=1.3)


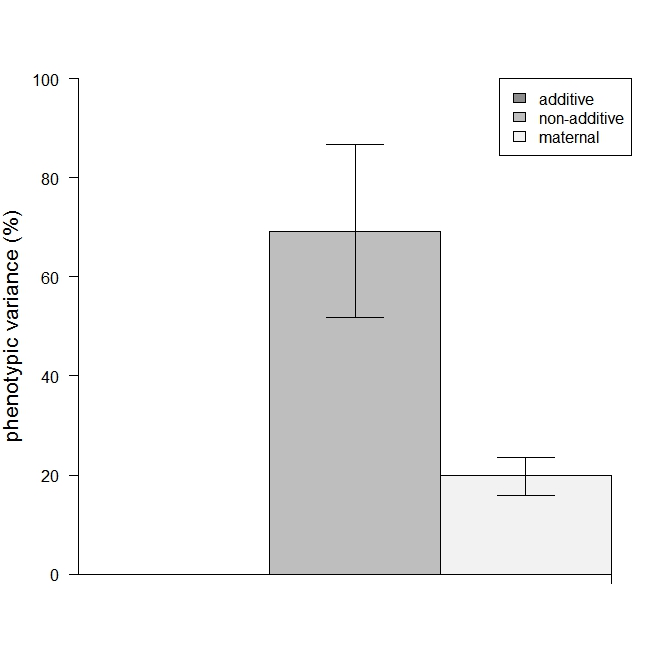


Figure S1. The additive genetic, non-additive genetic, and maternal effects underlying the phenotypic variance of the length at hatch for Chinook salmon (*Oncorhynchus tshawytscha*). The pseudo-value means are the tops of the shaded bars and the error bars cover the range of the 95% confidence intervals.
